# Supplementary material for: Completing the loop of the Late Jurassic–Early Cretaceous true polar wander event
Source: Nat Commun. 2024 Mar 12;15:2183. doi: 10.1038/s41467-024-46466-7 (PMC10933311; doi:10.1038/s41467-024-46466-7)
Supplement: Supplementary file 1 — Supplementary Information [file 41467_2024_46466_MOESM1_ESM.pdf]

Supplementary Information of

Completing the loop of the Late Jurassic–Early Cretaceous true polar wander event

Yifei Hou[1], Pan Zhao[1]*, Huafeng Qin[1], Ross N. Mitchell[1], Qiuli Li[1], Wenxing Hao[1], Min Zhang[2], Peter D. Ward[3], Jie Yuan[1], Chenglong Deng[1], Rixiang Zhu[1]

*Corresponding author. Email: panzhao@mail.iggcas.ac.cn;

[1] State Key Laboratory of Lithospheric Evolution, Institute of Geology and Geophysics, Chinese Academy of Sciences, Beijing 100029, China

[2] Key Laboratory of Earth and Planetary Physics, Institute of Geology and Geophysics, Chinese Academy of Sciences, Beijing 100029, China

[3] Department of Biology, University of Washington, Seattle, WA 98995, USA

*Corresponding author. Email: panzhao@mail.iggcas.ac.cn

This Supplementary Information (SI) contains four sections:

Supplementary Text
Supplementary Figs. 1 to 10
Supplementary Data 1 to 5
Supplementary References (1 to 41)

Supplementary Text

Geological setting and sampling

The North China craton (NCC) is centrally located in the East Asian continent (Supplementary Fig. 2a). Its basement is composed of Archean–Proterozoic metamorphic and sedimentary rocks [1, 2]. Amalgamation of the NCC with surrounding blocks was achieved through closure of the Paleo-Asian Ocean to the north in the late Paleozoic [3-5] and the Paleo-Tethys Ocean to the south in the Triassic [6-8]. In the Mesozoic, the northern margin of the NCC was characterized by the development of intracontinental rift basins that filled with Jurassic–Cretaceous volcanic rocks and terrestrial sediments [9, 10].

The Late Jurassic-Early Cretaceous stratigraphic sequence in the northern NCC comprises the Late Jurassic Tiaojishan Formation volcanic sequence and the Late Jurassic–Early Cretaceous Tuchengzi Formation sedimentary sequence [9] (Fig. 2a). The Tiaojishan Formation volcanic sequence consists predominantly of andesite, trachyandesite, and pyroclastic rocks with a few clastic interbeds, and is dated at 157 ± 3 Ma at the bottom and ca. 153 Ma on the top [11, 12]. The overlying Tuchengzi Formation is composed mainly of coarse-grained, purplish-red alluvial sediments, with volcanic rocks on the top [13, 14]. The Tuchengzi Formation is overlain by silicic volcanic rocks of the Early Cretaceous Zhangjiakou Formation [15] (Fig. 2a). Ages of these strata in our sampling region have been accurately determined. U–Pb and ^{40}Ar–^{39}Ar ages have been reported (Supplementary Data 3) from the top of the Tiaojishan Formation in the Chengde Basin (153 ± 1 Ma) [16], the Luanping Basin (153.8 ± 5.2 Ma) [17], and the Chicheng Basin (153 ± 1.1 Ma) [14]. Thus, based on those radiometric ages, deposition of the Tuchengzi Formation is considered to have started from ca. 153 Ma. To better constrain the depositional age of the Tuchengzi Formation, we collected two volcanic samples (TCZ-TA1, TCZ-TB1) from the top of the Tuchengzi

Formation from sections TCZ-TA and TCZ-TB, a red sandstone sample (TCZ-M1) and a volcanic sample (TCZ-M2) from the middle part of the Tuchengzi Formation from the TCZ-M section, and a volcanic sample (TJS-TC1) from the top of the Tiaojishan Formation from the TJS-TC section (Supplementary Data 3). Zircons U–Pb dating of volcanic samples were performed with Secondary ion mass spectrometry (SIMS) method, whereas that from sandstone samples were conducted with laser ablation–inductively coupled plasma–mass spectrometry (LA-ICP-MS) method.

Paleomagnetic samples were collected from the Chengde, Luanping, and Chicheng basins (Supplementary Figs. 2c-f). In the Chengde Basin (Supplementary Fig. 2c), 100 samples of grayish-green volcanic rocks were drilled from the top of the Tiaojishan Formation (section TJS-TA). In the Luanping Basin (Supplementary Fig. 2d), 45 samples of volcanic and pyroclastic rocks were also collected from the top of the Tiaojishan Formation (section TJS-TB). In the Chicheng Basin (Supplementary Figs. 2e, f), 50 samples of pyroclastic rocks were collected from the top of the Tiaojishan Formation (section TJS-TC), 120 red sandstone samples were got from the middle part of the Tuchengzi Formation (section TCZ-M). For the top of the Tuchengzi Formation, 148 samples were collected from sections TCZ-TA and TCZ-TB with a distance of ~10 km in the Chicheng Basin. All samples were drilled with a water-cooled portable driller and oriented using magnetic and solar compasses. There were no significant orientation differences between the two compasses. All core samples were cut into standard cylindrical specimens with 2.2 cm in length for paleomagnetic and anisotropy of magnetic susceptibility (AMS) measurements.

Zircon U–Pb dating results

For the two volcanic samples from the top of the Tuchengzi Formation, zircon grains are generally idiomorphic and up to 150 μm long and most grains presence of oscillatory zone in cathodoluminescence (CL) images which is the characteristic of magmatic zircons, as well as high

Th/U ratios (>0.2). At least 23 analyses of each sample provide a Concordia age of 141.6 ± 1.1 Ma (2σ (standard error), *n* = 28, MSWD (of concordance) = 0.5) and give consistent $^{206}Pb/^{238}U$ weighted mean ages of 141.8 ± 1.1 Ma (2σ, *n* = 28, MSWD = 0.9) for sample TCZ-TA1 and a Concordia age of 140.9 ± 1.2 Ma (2σ, *n* = 23, MSWD (of concordance) = 2.1) as well as a weighted average $^{206}Pb/^{238}U$ age of 141.1 ± 1.7 Ma (2σ, *n* = 23, MSWD = 1.6) for sample TCZ-TB1, respectively (Figs. 2b, c; Supplementary Data 2).

For the volcanic sample (TSJ-TC1) from the top of Tiaojishan Formation, most of zircon grains are small and about 50 μm long (Fig. 2d) but also has the characteristic of magmatic zircons, oscillatory zone in CL images and high Th/U ratios (>0.2). Thirty-nine concordant ages have been obtained, results of which are listed in Supplementary Data 2. Excluding the two Precambrian and one Paleozoic zircon grains, the other 36 grains show two age groups. In the Chicheng-Jingxi, Luanping-Chengde Basins, radiometric dating results showed that the Tiaojishan Formation volcanic rocks are younger than ~157Ma [9, 10]. Therefore, the older age group may represent inherited zircons. We choose eight youngest eight zircon grains that yield a concordia age of 152.6 ± 2.2 Ma (2σ, *n* = 8, MSWD of concordance = 0.8) as well as a weighted average $^{206}Pb/^{238}U$ age of 152.9 ± 2.5 Ma (2σ, *n* = 8, MSWD = 0.3, Fig. 2d), which is taken as the eruption age. This SIMS zircon U-Pb age is consistent with previous research by SHRIMP method in the same basin (Fig. 2a). However, more precise CA-ID-TIMS U-Pb dating of the Tiaojishan Formation from the Jianchang Basin in the eastern part of the NCC yielded an age of 160.245 ± 0.045 Ma which is much older than our result[19]. Previous magmatic studies have shown that the magmatic activity of Tiaojishan Formation experienced westward migration, resulting in westward younging trend of eruption ages of the Tiaojishan Formation in different basins [18]. As the Jianchang Basin is located ~340 km east of our cross section, we argue the age difference resulted from magmatic migration

of the Tiaojishan volcanism. Besides, in the Jianchang Basin, SIMS zircon U-Pb dating was also performed from the same cross section, which gave an age of 159.5 ± 2.3 Ma [19], which is consistent with the CA-ID-TIMS age. Therefore, despite different precision, SIMS and CA-ID-TIMS methods can give consistent results without obvious difference. In conclusion, we consider that the eruption age of the TJS-TC section in the Chicheng Basin should be ca. 153 Ma.

The youngest age group of detrital zircons can constrain the maximum depositional age for clastic sediments. For the red sandstone sample collected from the middle part of the Tuchengzi Formation (sample TCZ-M1), two youngest zircon grains give an age of 151 Ma which can constrain the depositional age of sampling section in the range of 141 and 151 Ma (Fig. 3e). The pyroclastic rock sample (TCZ-M2) was collected below the sample TCZ-M1, therefore, age of this sample must be younger than 151 Ma. Actually, the SIMS zircon dating results yielded continuous zircon ages from 166.4 ± 4.7 to 147.1 ± 3.9 Ma (Fig. 2f; Supplementary Data 2 and 3), indicating abundant inherited zircons. Therefore, we use the youngest three ages to calculate a weighted mean $^{206}Pb/^{238}U$ age of 147.5 ± 4.5 Ma (2σ, n = 3, MSWD = 0.03; Fig. 2g). In this case, we assume that the best estimate of the TCZ-M section should be ca. 147 Ma.

Rock magnetism

All the selected samples can be divided into two types of magnetic carriers according to hysteresis loop and the isothermal remanent magnetization (IRM) measurements. For volcanic and volcanoclastic rocks (TJS-TC8, TCZ-TA51, TCZ-TB125), the hysteresis loops for selected specimens reach saturation above 0.5 T with low coercivity at 7.2–18 mT (Supplementary Figs. 4a, c, d). The IRM acquisition curves increase rapidly before 100 mT and reach 100% saturation at ~300 mT (Supplementary Figs. 4e, g, h). The gradient curves of IRM acquisition display a predominant low-coercivity component (37.2–41.7 mT) with a subordinate high coercivity

component (316.2–354.8 mT) (Supplementary Figs. 4i, k, l). Based on these features, we suggest that magnetite is the main magnetic remanence carrier for volcanic and volcanoclastic specimens from the top of the Tiaojishan Formation, the top of the Tuchengzi Formation.

For the red sandstone samples from the middle part of the Tuchengzi Formation (TCZ-M24), the hysteresis loop (Supplementary Fig. 4b) shows a pronounced wasp-waisted shape, suggesting the existence of both soft (magnetite) and hard (hematite) magnetic minerals [20]. The IRM acquisition curve shows gradual increases of magnetization and less than half-saturation at 0.3 T without saturation at 1.5 T (Supplementary Fig. 4f). The gradient curve of IRM acquisition (Supplementary Fig. 4j) shows a dominant high coercivity component with value at ~1 T and two subordinates low coercivity component (50.1 mT) and middle coercivity component (316.2 mT). Therefore, we can conclude that hematite is the main magnetic carrier for the red sandstone samples with some portions of magnetite.

Fold and Reversal tests

For the top of the Tiaojishan Formation (153 Ma) after rotation, the fold test of McElhinny [21] is positive at 99% confidence as n=162, ks/kg=3.37 > F (322, 322) =1.32 and the fold test of McFadden [22] is also positive at 99% confidence as n=162, in-situ ξ_2=70.8 > statistical threshold ξ=20.9 > tilt-corrected ξ_2=1.7 at 99% confidence level. Both the reversal tests of MaFadden and McElhinny [23] and the bootstrap reversal test [24] for the section TJS-TC give negative feedback (Supplementary Fig. 10a), we consider that may be caused by relative tectonic rotation and/or much less the reversed specimens than the normal ones. Nevertheless, as the data pass the fold test, we still consider that samples from the top of the Tiaojishan Formation record primary magnetization.

For the middle part of the Tuchengzi Formation (ca. 147 Ma), the reversal test of MaFadden and McElhinny [23] is positive at level-C as the angle between the mean directions of normal (D/I=7.9°/38.2°, k=15.5, n=92) and reversal (D/I=201.2°/-39.8°, k=19.5, n=11) samples γ=10.5° < $\gamma_{critical}$=11.6°. The bootstrap reversal test [24] shows overlapped X and Z components (Supplementary Fig. 10b). Although the Y components of normal and reversed directions are not overlapped, they are very closed (Supplementary Fig. 10b). Therefore, considering results of both reversal tests, the characteristic remanent magnetization (ChRM) directions of the middle part of the Tuchengzi Formation should be primary magnetization.

Combining the two sections from the top of the Tuchengzi Formation after rotation, the fold test of McElhinny [21] is positive at 99% confidence as n=104, ks/kg=1.89 > F (206, 206) =1.37 and the fold of McFadden[22] is also positive at 99% confidence as n=104, in-situ ξ_2=57.6 > statistical threshold ξ=16.8 > tilt-corrected ξ_2=1.3 at 99% confidence level. Only section TCZ-TA display both normal and reversed polarities and the reversal test of MaFadden and McElhinny [23] is positive at level-B as the angle between the mean directions of normal (D/I=8.2°/53.8°, k=40.5, n=35) and reversal (D/I=180.2°/-45.5°, k=47.3, n=6) samples γ=9.7° < $\gamma_{critical}$=9.9°. The bootstrap reversal test [24] shows that both X and Z components of normal and reversed directions are not overlapped (Supplementary Fig. 10b), indicating a negative reversal test. However, considering the positive fold tests and positive reversal test of MaFadden and McElhinny [23], the ChRM directions of the top of the Tuchegzi Formation should be primary magnetization.

Paleomagnetic poles

Controversy about the “monster shift” is mainly resulted from different understanding of the paleomagnetic poles in the age of 147–145 Ma. Kent and Irving [25] (hereafter K&I) combined three ca. 145 Ma poles, the 147 Ma Swartruggens-Bumbeni kimberlite pole from southern Africa [26, 27],

the 146 Ma Ithaca kimberlite pole from North America [28], and the 144 Ma Hinlopensretetsills pole from Svalbard [29], to form a coherent cluster at ca.145 Ma, which show a significant polar shift comparing with ca. 160 and ca. 155 Ma poles, arguing for a Late Jurassic "monster shift". However, Kulakov et al. [30] argued that the Hinlopenstretet pole is younger than previously thought due to recent dating results and the Swartuggens-Bumbeni pole cannot be used as a reference pole for constraining the Jurassic APWP due to possible influence of local structure as well as controversial age. Although the emplacement age of the Ithaca dykes was robustly determined at ca. 146 Ma by U–Pb dating of perovskite [31], Kulakov et al. [30] suspected that the data did not average out the paleosecular variation considering the low angular dispersion of paleomagnetic directions. In this case, all three available ca. 145 Ma paleomagnetic poles are not reliable. Meanwhile, Kulakov et al. [30] have recently reported a ca. 147 Ma paleomagnetic pole from southwest Greenland, which is close to the ca. 150 Ma poles, supporting for a steady polar motion with rates of ~0.7° Mry^{-1}, two to three times slower than the "monster shift". Based on these evidence, they argued that the monster shift may be an artifact of paleomagnetic and geochronological data [30].

For the new ca. 147 Ma pole from Greenland [30], the paleomagnetic data are robust, whereas the age of sampled dykes should be treated with caution. Dating of sampled dykes in southwestern Greenland yielded two groups of ages at 149–156 Ma with Rb-Sr and U–Pb methods and 141–136 Ma with ^{40}Ar–^{39}Ar method. Usually, as the U–Pb system has a high block temperature, the U–Pb ages can better represent the emplacement age of the studied dykes. On the contrary, the ^{40}Ar–^{39}Ar system has a relative low close temperature of 300–500°C that could be easily affected by thermal or tectonic events. Therefore, the 149–156 Ma may be the best estimate for the age of studied dykes, whereas the 141–136 Ma ^{40}Ar-^{39}Ar ages may represent latter reset. Actually, the Greenland poles indeed overlaps with the 150 and 154 Ma poles [30], which may also indicate that emplacement

of these dykes can be estimated to actually be in the range of 149–156 Ma. We noticed that the ca. 146 Ma pole from the Ithaca dykes consists of both normal and reverse polarities and passes a reversal test. Therefore, the acquisition of magnetization should be long enough to average out the paleosecular variation. Therefore, we think that the Ithaca pole should be reliable and can be used to construct the Jurassic APWP, which support the “monster shift” during the Jurassic Period.

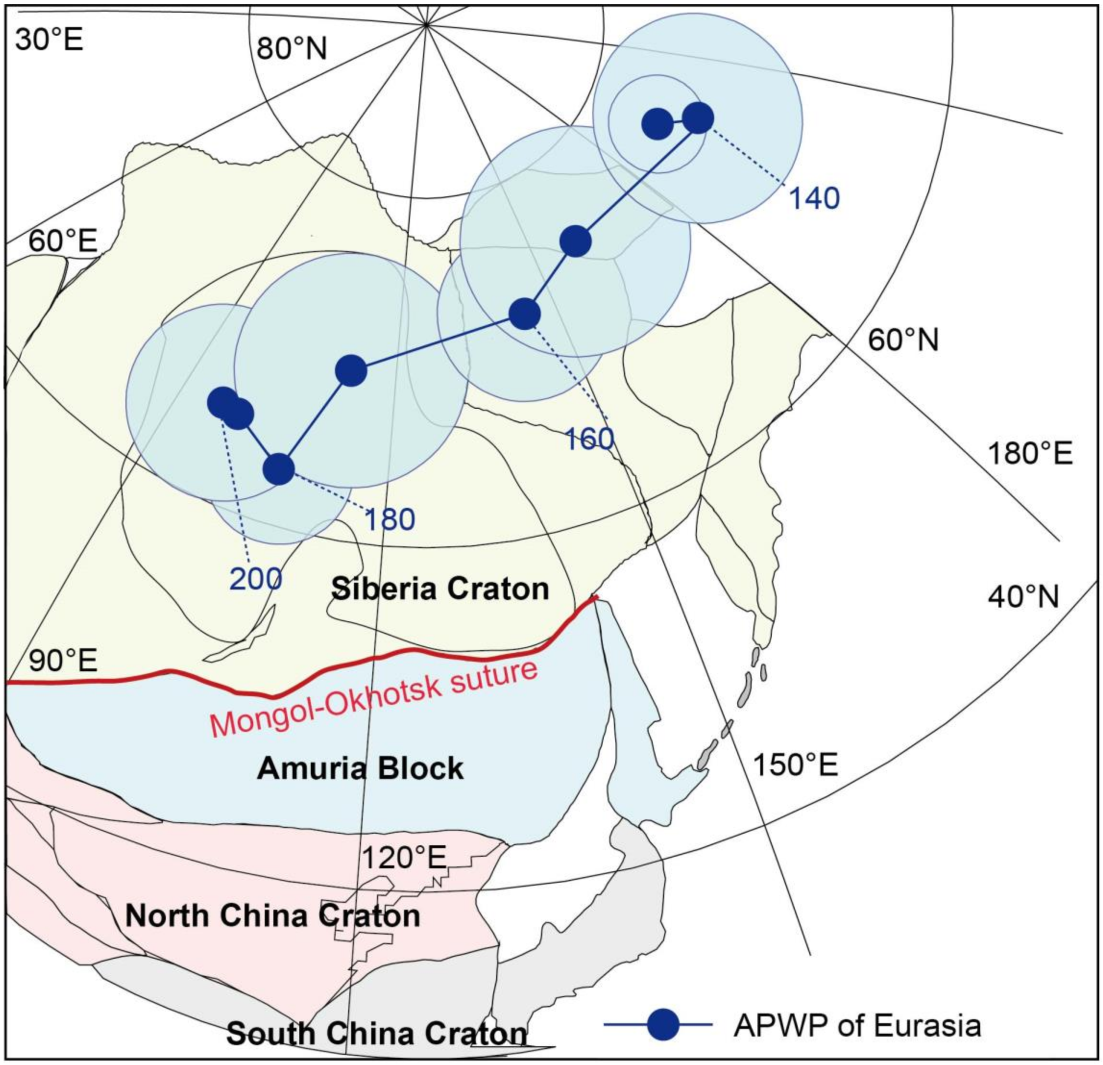

Supplementary Fig. 1 | Tectonic sketch map of East Asia. Map showing main blocks with running-mean APWPs with window length 10 Myr for Eurasia after Besse and Courtillot [32].

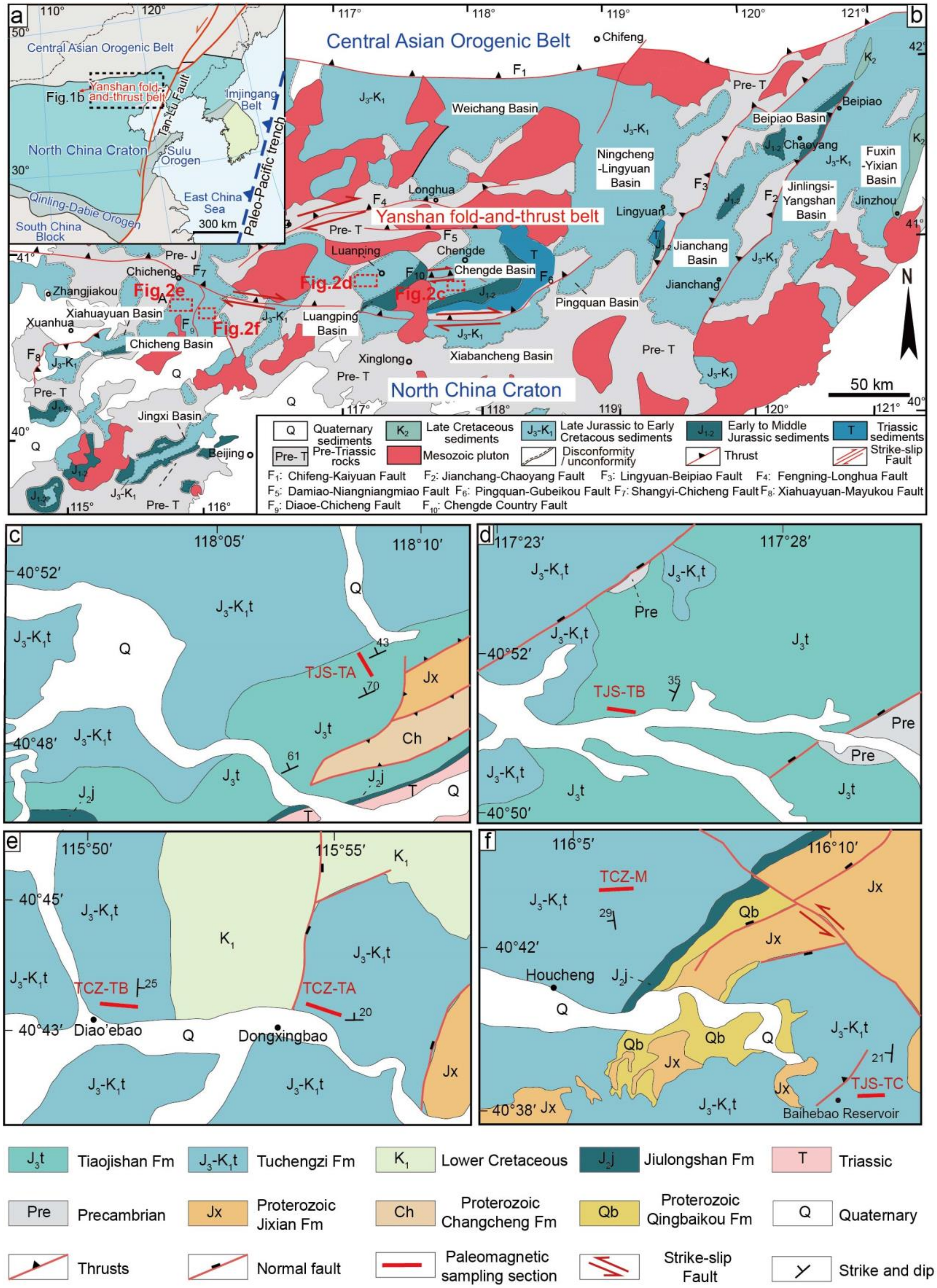
a
Central Asian Orogenic Belt
Yanshan fold-and-thrust belt
Fig.1b
Tan-Lu Fault
Imjingang Belt
North China Craton
Sulu Orogen
East China Sea
Paleo-Pacific trench
Qinling-Dabie Orogen
South China Block
300 km
b
Central Asian Orogenic Belt
Chifeng
Weichang Basin
Beipiao Basin
Beipiao
Chaoyang
Ningcheng -Lingyuan Basin
Fuxin -Yixian Basin
Jinlingsi-Yangshan Basin
Jinzhou
Longhua
Lingyuan
Yanshan fold-and-thrust belt
Luanping
Chengde
Chengde Basin
Jianchang Basin
Jianchang
Pingquan Basin
Chicheng
Zhangjiakou
Xiahuayuan Basin
Xuanhua
Chicheng Basin
Luangping Basin
Xinglong
Xiabancheng Basin
North China Craton
Jingxi Basin
Beijing
Fig.2c
Fig.2d
Fig.2e
Fig.2f
50 km
Quaternary sediments
Pre-Triassic rocks
Late Cretaceous sediments
Mesozoic pluton
Late Jurassic to Early Cretacous sediments
Disconformity / unconformity
Early to Middle Jurassic sediments
Thrust
Triassic sediments
Strike-slip Fault
F1: Chifeng-Kaiyuan Fault F2: Jianchang-Chaoyang Fault F3: Lingyuan-Beipiao Fault F4: Fengning-Longhua Fault
F5: Damiao-Niangniangmiao Fault F6: Pingquan-Gubeikou Fault F7: Shangyi-Chicheng Fault F8: Xiahuayuan-Mayukou Fault
F9: Diaoe-Chicheng Fault F10: Chengde Country Fault
c
TJS-TA
d
TJS-TB
e
TCZ-TB
TCZ-TA
Diao'ebao
Dongxingbao
f
TCZ-M
Houcheng
TJS-TC
Baihebao Reservoir
Tiaojishan Fm
Tuchengzi Fm
Lower Cretaceous
Jiulongshan Fm
Triassic
Precambrian
Proterozoic Jixian Fm
Proterozoic Changcheng Fm
Proterozoic Qingbaikou Fm
Quaternary
Thrusts
Normal fault
Paleomagnetic sampling section
Strike-slip Fault
Strike and dip

Supplementary Fig. 2 | Geological maps of the sampling region. (a) Sketched tectonic map of East Asia showing main cratons/blocks and orogenic belts. **(b)** Geological map of the north margin of the NCC, showing locations of Mesozoic basins with locations of our sampling regions. Detailed geological maps for sampling basins, **(c)** Chengde Basin, **(d)** Luanping Basin, **(e and f)** Chicheng Basin with locations of paleomagnetic sampling sections.

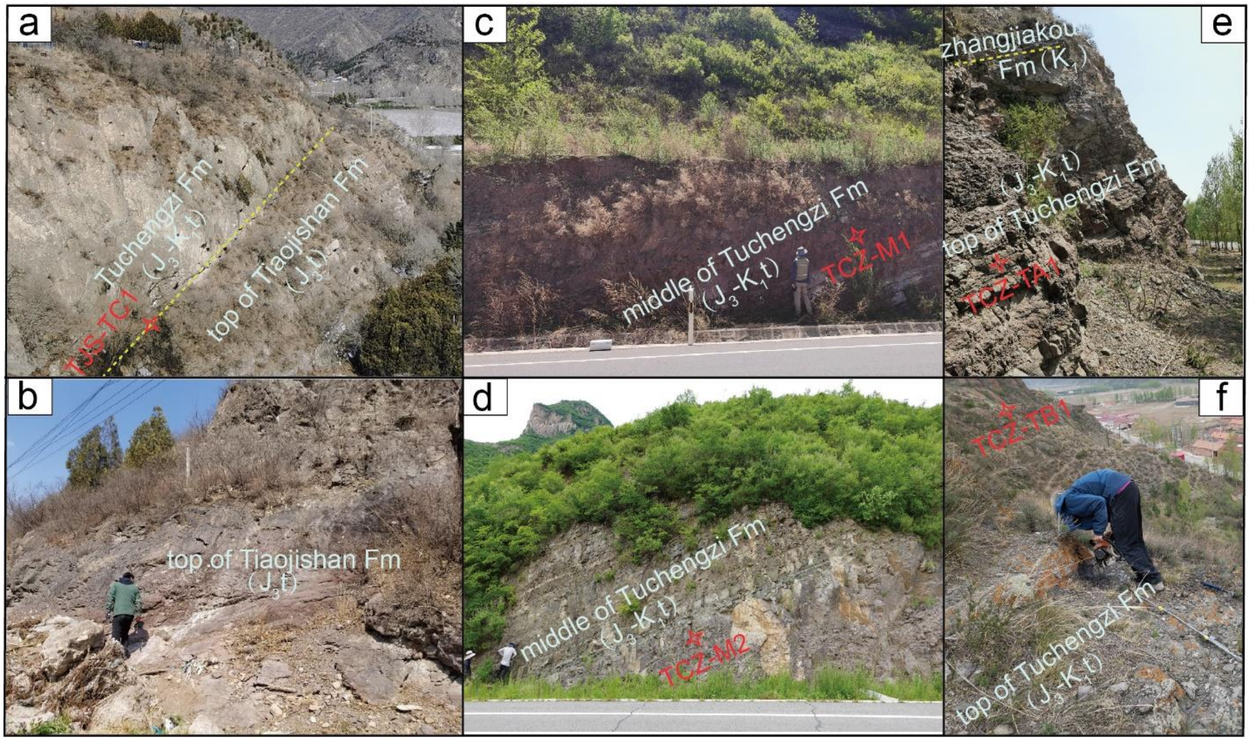

Supplementary Fig. 3 | Outcrops of sampled Tiaojishan and Tuchengzi Formations. (a) The Tuchengzi Formation conformably overlies the Tiaojishan Formation in Chicheng Basin (section TJS-TC) and U–Pb dating sample (TJS-TC1) from the top of the Tiaojishan Formation. **(b)** Section TJS-TB in the top of Tiaojishan Formation from Luanping Basin. **(c)** Red sandstones of the middle Tuchengzi Formation (section TCZ-M) and U–Pb dating sample (TCZ-M1). **(d)** The section TCZ-M and pyroclastic rock sample (TCZ-M2) for U–Pb dating from the middle of the Tuchengzi Formation. **(e)** The Zhangjiakou Formation overlies Tuchengzi Formation in Chicheng Basin (section TCZ-TA) with an unconformity or paraconfromity, with the dating sample (TJS-TA1) being from the local top of the Tuchengzi Formation. **(f)** Section TCZ-TB in the top of Tuchengzi Fm with U–Pb dating sample (TCZ-TB1) from Chicheng Basin.

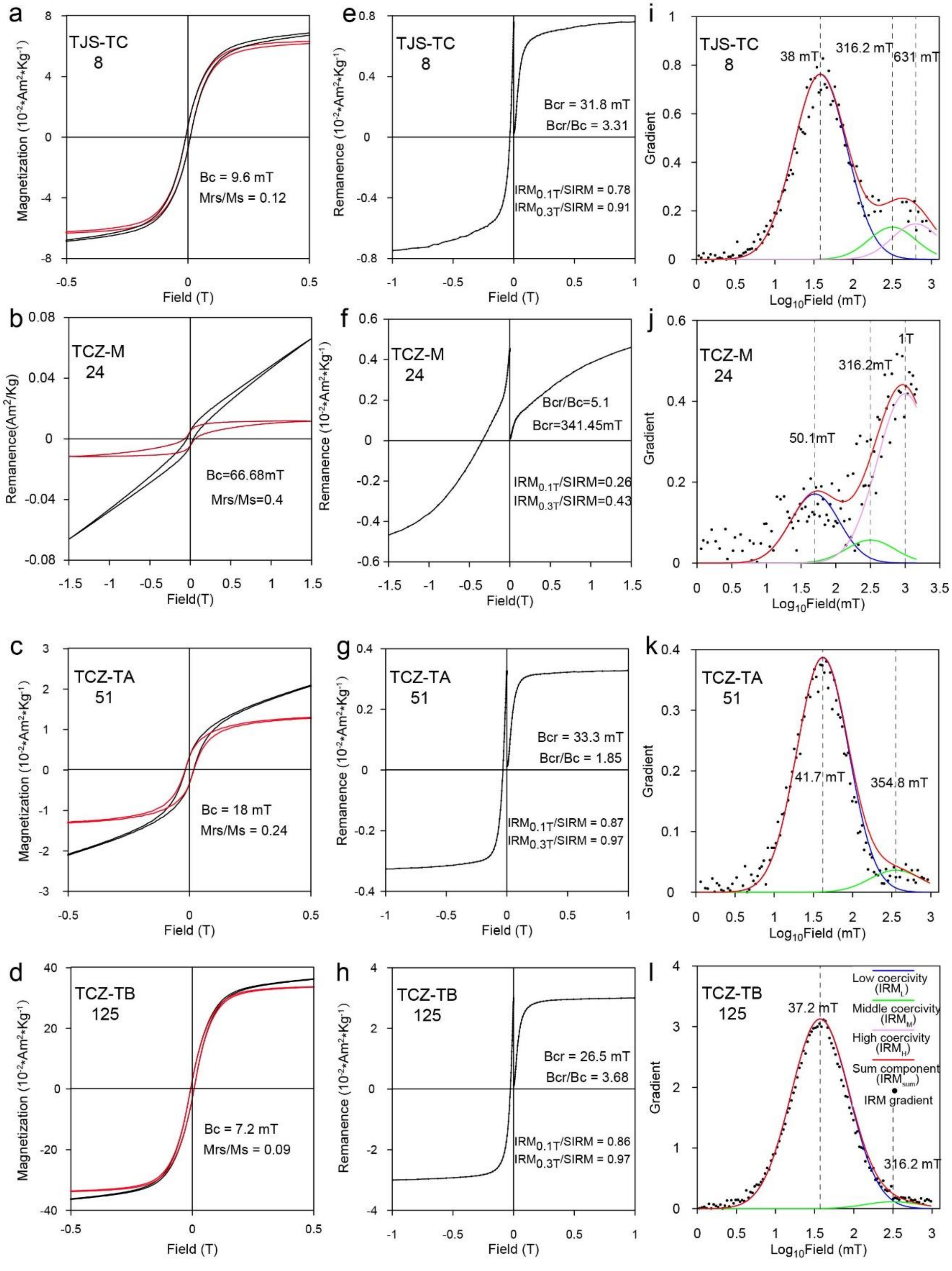

Supplementary Fig. 4 | Rock magnetic results. (a–d) Hysteresis loops before (black line) and after (red line) high-field slope correction, which were measured in fields cycled between ± 1.5 T and cut off at ± 0.5 T for reasons of clarity. **(e–h)** Isothermal remanent magnetization (IRM)

acquisition and back-field demagnetization curves. **(i–l)** Component analysis of coercivity distributions.

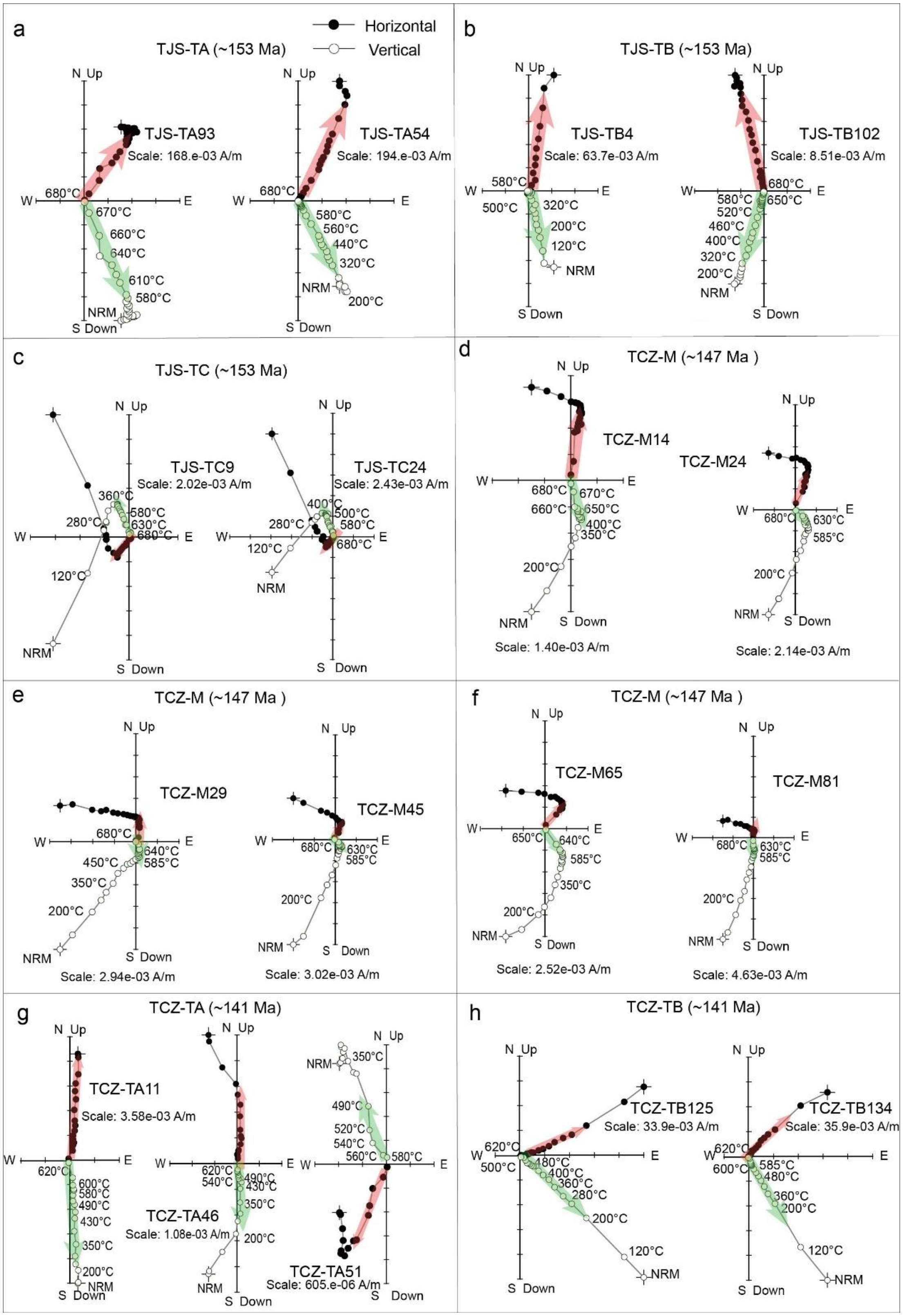

Supplementary Fig. 5 | Typical orthogonal vector plots for demagnetization results.

Magnetic directions are plotted in tilt-corrected coordinates for representative specimens from

the (a) section TJS-TA, (b) section TJS-TB, (c) section TJS-TC, (d, e, f) section TCZ-M, (g) section TCZ-TA, and (h) section TCZ-TB. Black/white circles represent vector endpoints projected onto the horizontal/vertical plane. Numbers on the plots show temperature steps in °C. NRM: natural remanent magnetization.

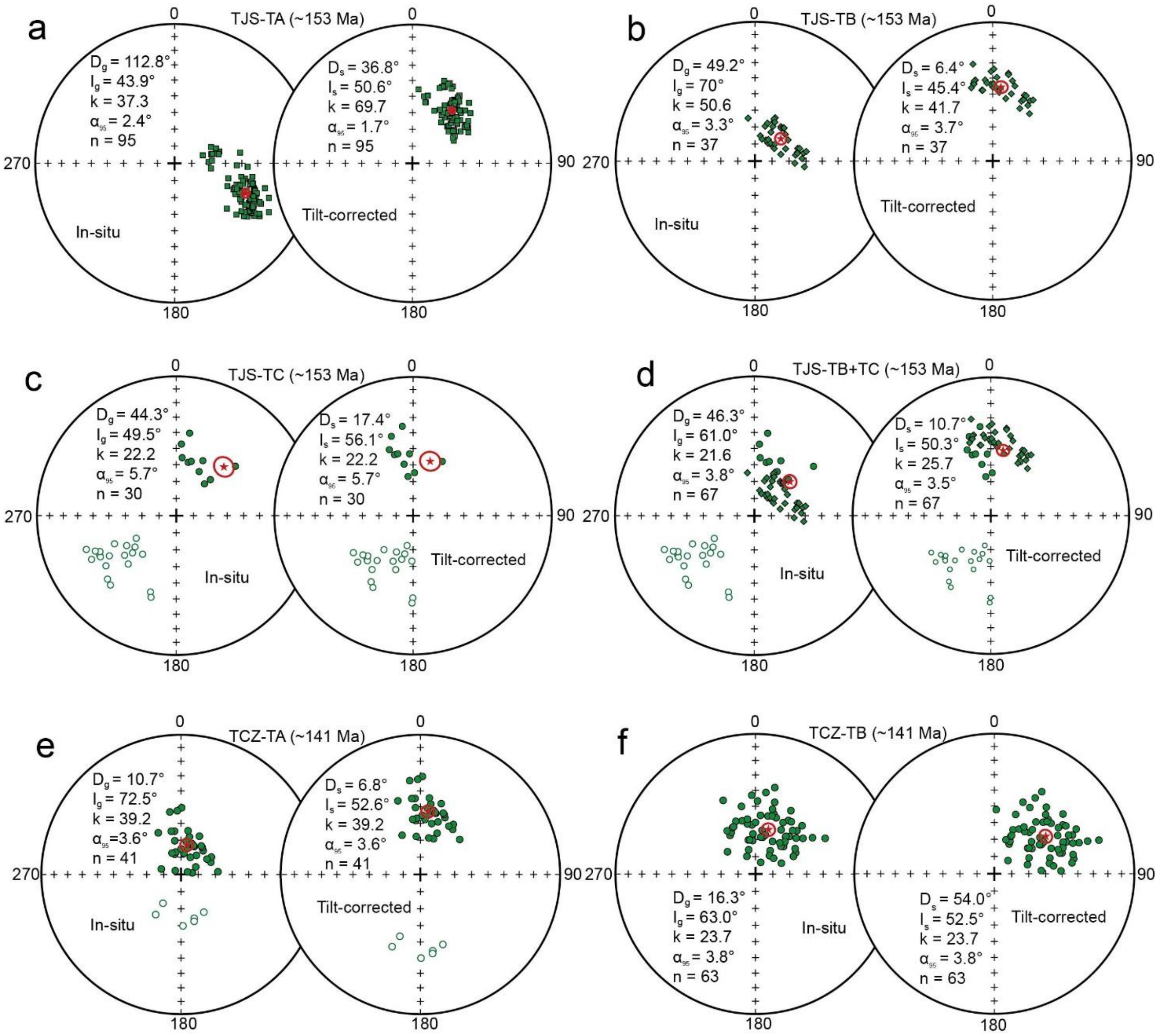

Supplementary Fig. 6 | Paleomagnetic results. Equal-area projections of the paleomagnetic directions of the high-temperature components (green), showing mean directions from each section (red). Statistical results, for section TJS-TB **(a)**, section TJS-TC **(b)**, combined specimens from two sections of TJS-TB and TJS-TC **(c)**, and section TJS-TA **(d)** from the top of the Tiaojishan Formation (ca. 153 Ma), for section TCZ-TA **(e)** and section TCZ-TB **(f)** from the top of the Tuchengzi Formation (ca. 141 Ma). Results were calculated with 95% confidence limits.

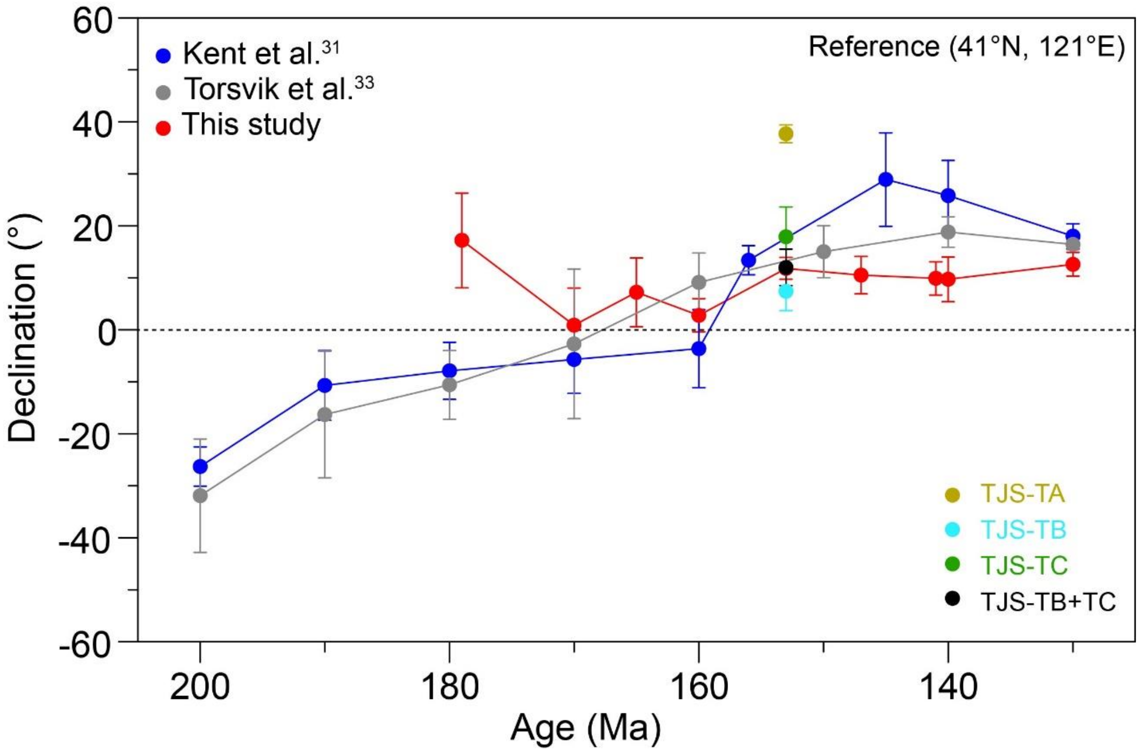

Supplementary Fig. 7 | Comparison of Jurassic-Early Cretaceous Declination data. Declination data from reported paleopoles of NCC and two computed global APWPs by Kent et al.[31] and Torsvik et al.[33] between 200 Ma and 130 Ma. All declinations were calculated with reference point at 41°N, 121°E. Error bars are calculated with A_{95} of each paleopole.

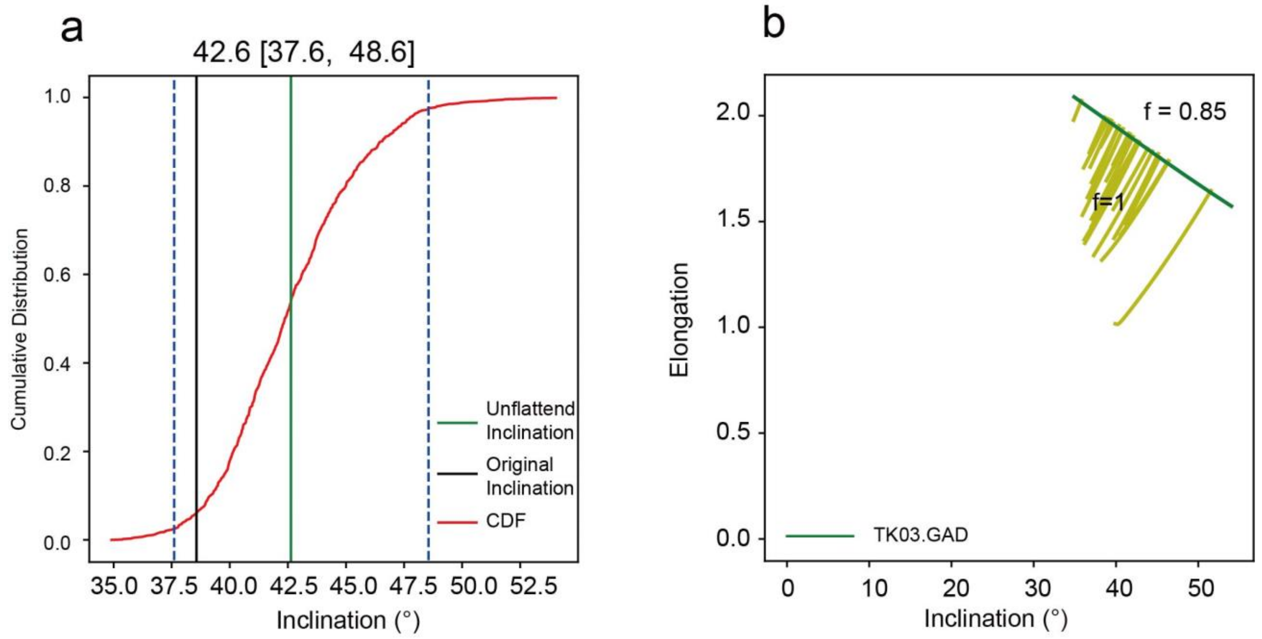

Supplementary Fig. 8 | Inclination shallowing correction. (**a**) E/I analyses of the 103 ChRM directions. (**b**) Correction for the inclination shallowing of the 103 ChRM directions from the section TCZ-M using the E/I method, which gives a flattening factor *f* = 0.85.

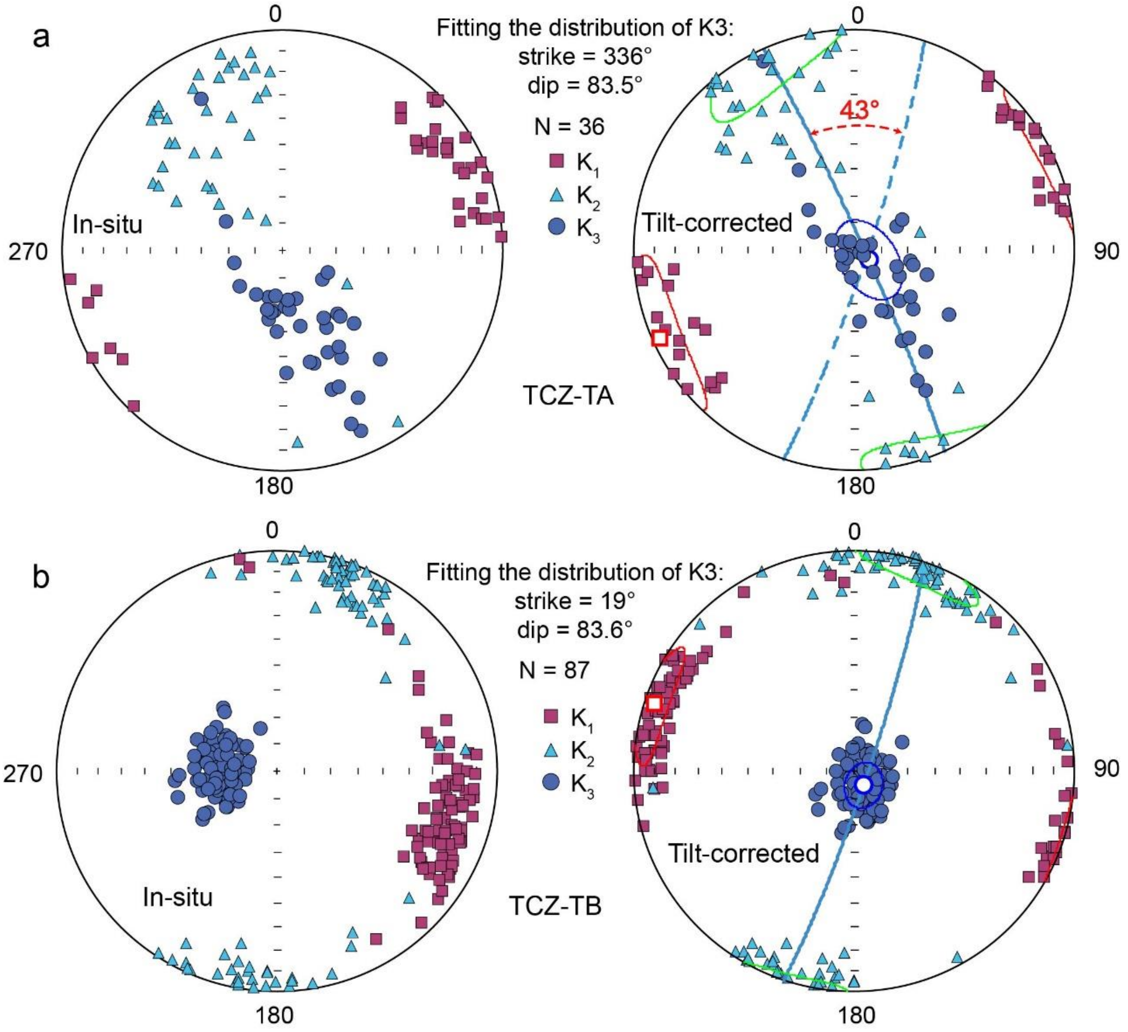

Supplementary Fig. 9 | Stereographic projections of the AMS results. The AMS results of the **(a)** TCZ-TA and **(b)** TCZ-TB sections in geographical (stratigraphic) coordinates. Light blue line, the plane of great-circle fitting the distribution of K_3. Red square, the pole to plane of fitting the distribution of K_3 with 95% confidence limit.

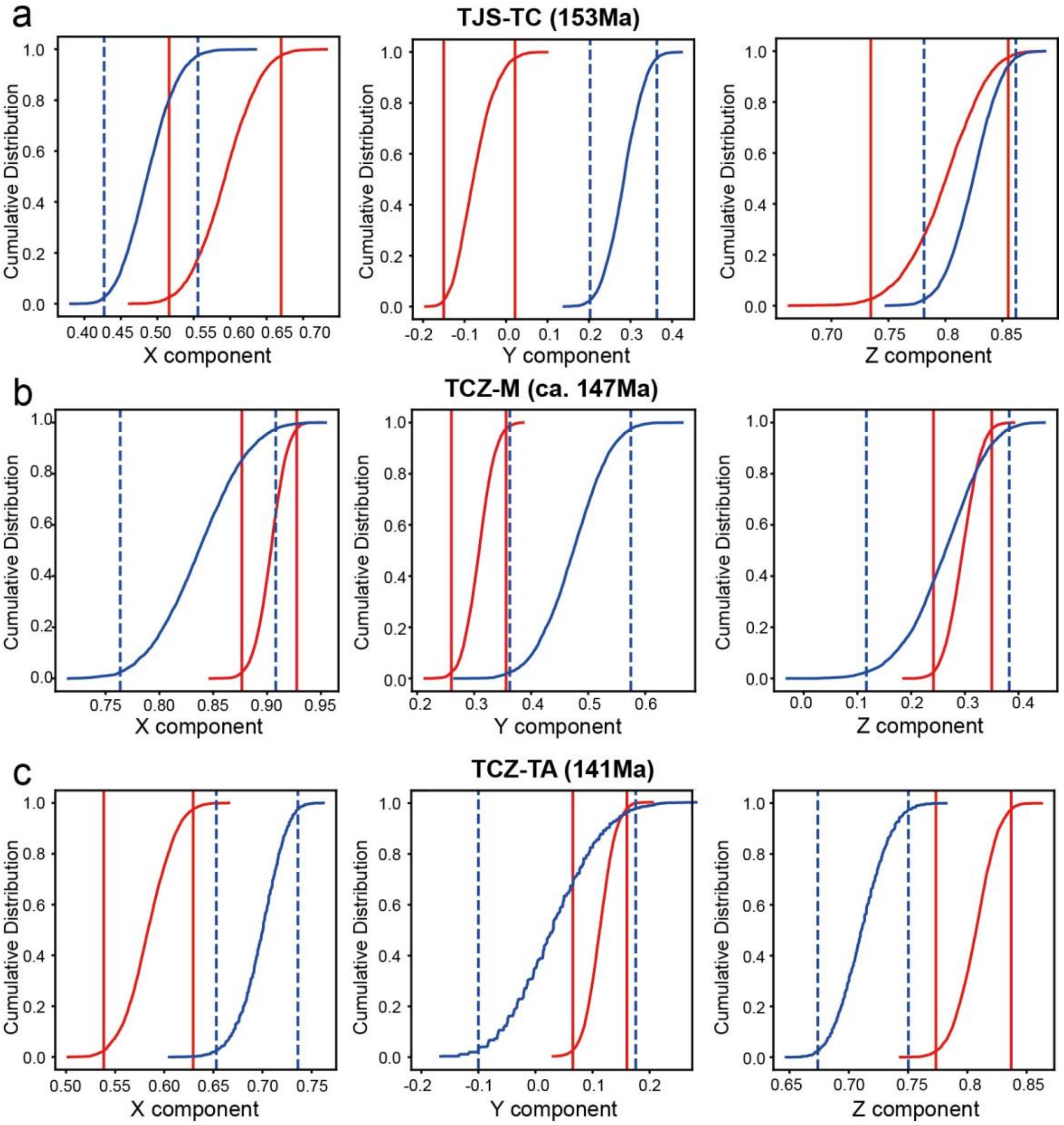

Supplementary Fig. 10 | The results of bootstrap reversal test. From top to bottom are the results of bootstrap reversal test from sections TJS-TTC **(a)**; TCZ-M **(b)**; and TCZ-TA **(c)**. Reversed polarity directions have been inverted to their antipodes to test a common mean for the normal (blue) and reversed (red) magnetization directions.

Supplementary Table 1.

Jurassic to early Cretaceous Running Mean Appareent polar wander paths (APWPs) and paleomagnetic poles for different plates rotated to northwest Africa coordinates and Eurasia coordinates, shown in Fig. 1 and Fig. 4.

(1) Kent and Irving [25] North America APWP rotated to northwest Africa coordinates.

Age (Ma)	Long. (°E)	Lat. (°N)	A_{95} (°)
130	264.2	49.5	2.4
140	264.4	42.8	6.8
145	266.2	38.6	9
160	259.1	66.4	7.5
170	258.7	66.5	6.5
180	269.7	65.4	5.5
190	270.3	66.9	6.7
200	238.1	71.9	3.8

(2) Torsvik et al. [33] South Africa APWP rotated to northwest Africa coordinates.

Age (Ma)	Long. (°E)	Lat. (°N)	A_{95} (°)
130	258.6	50.6	2.8
140	259.7	47.4	6
150	254.2	54.6	6.4
160	252.7	56.8	5.1
170	250.6	57.3	4.6
180	254.9	63.6	3.4
190	252.2	68.2	2.9
200	236.6	70.1	2.8

(3) Muttoni et al. [34] Adria APWP rotated to northwest Africa coordinates.

Age (Ma)	Long. (°E)	Lat. (°N)	A_{95} (°)
143	263.5	45.3	3.1
148	265.4	37.6	4.1
158	266.5	56.8	4.3
158	254.9	64.8	3.7
183	271.5	79.5	3.2

(4) Fu et al. [35] South America paleomagnetic poles rotated to northwest Africa coordinates.

Age (Ma)	Long. (°E)	Lat. (°N)	A_{95} (°)
152.8	265	51.1	10.8
165.8	265.8	59.9	7.6

(5) Kent et al. [31] North America APWP rotated to Eurasia coordinates.

Age (Ma)	Long. (°E)	Lat. (°N)	A_{95} (°)
130	198.8	76.5	2.4

140	209.2	70.1	6.8
145	214.5	66.9	9
156	185.4	79.5	2.8
160	114	73.7	7.5
170	112	71.1	6.5
180	104.6	74.2	5.5
190	99.5	73.5	6.7
200	98.6	61.3	3.8

(6) Torsvik et al. [33] North America APWP rotated to Eurasia coordinates.

Age (Ma)	Long. (°E)	Lat. (°N)	A_{95} (°)
130	182.4	77.1	1
140	181.7	75.3	2.9
150	162.3	75.7	5
160	138	72.9	5.7
170	117.8	67.7	14.4
180	107.2	68.5	6.6
190	101.8	66.6	12.2
200	99.7	57.7	10.9

(7) Muttoni and Kent [36] Adria APWP rotated to Eurasia coordinates.

Age (Ma)	Long. (°E)	Lat. (°N)	A_{95} (°)
143	198.8	72	3.1
148	215.2	66.5	3.1
158	154.5	78.1	4.3
160	107.7	74.1	4

(8) North China craton APWP rotated to northwest Africa coordinates by rotation parameter (353.5, 1.2, 32.9).

Age (Ma)	Long. (°E)	Lat. (°N)	A_{95} (°)
179	273.8	49.6	5.5
170	265.0	56.3	7.1
165	260.2	51.9	6.6
160	269.2	49.7	3.2
155	239.4	40.2	5.1
153	260.9	44.5	2.1
147	266.9	39.1	3.6
141	260.7	47.7	3.2
140	255.9	52.4	4.3
130	251.4	52.3	2.3

(9) North China craton APWP rotated to Eurasia coordinates by rotation parameter (93.1, 71.4, -31.6).

Age (Ma)	Long. (°E)	Lat. (°N)	A_{95} (°)

179	214.9	78.7	5.5
170	169.3	79.4	7.1
165	179.6	74.7	6.6
160	202.7	77.1	3.2
155	175.5	56.3	5.1
153	197.4	69.6	2.1
147	215.1	67.0	3.6
141	191.0	72.0	3.2
140	172.6	72.8	4.3
130	167.7	70.6	2.3

Abbreviations: Lat., Long.: latitude and longitude of mean paleopole; A_{95}, 95% confidence limit. (1), (3), (5), (6), (7) Using rotation parameters in Kent and Irving [25]. (2), (4) Using rotation parameters in Torsvik et al. [33].

Supplementary Table 2.

SIMS and LA-ICP-MS zircon U–Pb dating results of volcanic rocks and sandstone from the Tiaojishan and Tuchengzi Formations in north margin of the North China Craton.

	Radiogenic Isotope Ratios					Isotopic Ages(Ma)						
**Sample/ spot #**	**$^{207}Pb/^{235}U$**	**±σ %**	**$^{206}Pb/^{238}U$**	**±σ %**	**rho**	**$^{207}Pb/^{235}U$**	**±σ**	**$^{206}Pb/^{238}U$**	**±σ**	**[U] ppm**	**[Th] ppm**	**Th/U meas**
						**TCZ-TA1**						
TCZ-TA-1	0.136	13.4	0.0217	2.0	0.1	129.69	16	138	3	166	73	0.4
TCZ-TA-2	0.147	5.3	0.0221	2.1	0.4	139.33	7	141	3	79	65	0.8
TCZ-TA1-3	0.154	6.4	0.0220	1.8	0.3	145.23	9	140	2	246	119	0.5
TCZ-TA1-4	0.151	3.8	0.0222	2.1	0.5	142.94	5	142	3	163	41	0.3
TCZ-TA1-5	0.135	5.0	0.0213	3.3	0.7	128.31	6	136	4	232	166	0.7
TCZ-TA1-6	0.154	5.2	0.0216	1.8	0.3	145.80	7	138	2	113	65	0.6
TCZ-TA1-7	0.166	7.3	0.0226	2.1	0.3	156.22	11	144	3	188	158	0.8
TCZ-TA1-8	0.143	5.7	0.0221	2.1	0.4	135.28	7	141	3	155	103	0.7
TCZ-TA1-9	0.139	5.9	0.0217	2.4	0.4	131.92	7	138	3	113	58	0.5
TCZ-TA1-10	0.155	4.6	0.0222	2.0	0.4	146.26	6	142	3	234	192	0.8
TCZ-TA1-11	0.150	4.8	0.0218	2.0	0.4	142.00	6	139	3	165	68	0.4
TCZ-TA1-12	0.149	9.8	0.0228	2.0	0.2	140.90	13	145	3	213	42	0.2
TCZ-TA1-13	0.163	6.9	0.0226	1.8	0.3	153.53	10	144	3	105	47	0.4
TCZ-TA1-14	0.352	3.7	0.0486	1.6	0.4	306.16	10	306	5	500	113	0.2
TCZ-TA1-15	0.147	2.3	0.0223	1.5	0.7	139.42	3	142	2	1356	1259	0.9
TCZ-TA1-16	0.151	3.4	0.0227	1.8	0.5	142.64	5	145	3	439	146	0.3
TCZ-TA1-17	0.147	6.4	0.0227	1.7	0.3	138.95	8	145	2	200	95	0.5
TCZ-TA1-18	0.150	7.7	0.0220	2.0	0.3	141.80	10	140	3	117	110	0.9
TCZ-TA1-19	0.132	8.8	0.0223	2.1	0.2	125.50	11	142	3	115	90	0.8
TCZ-TA1-20	0.148	13.7	0.0232	2.1	0.2	139.85	18	148	3	207	192	0.9
TCZ-TA1-21	0.150	5.0	0.0230	2.6	0.5	141.94	7	146	4	146	76	0.5
TCZ-TA1-22	0.155	4.3	0.0214	2.1	0.5	146.09	6	136	3	73	43	0.6
TCZ-TA1-23	0.175	4.0	0.0229	2.2	0.5	163.76	6	146	3	143	109	0.8

TCZ-TA1-24	0.148	5.2	0.0225	1.7	0.3	139.86	7	144	2	206	168	0.8
TCZ-TA1-25	0.149	4.2	0.0224	1.9	0.5	140.78	6	143	3	234	101	0.4
TCZ-TA1-26	0.145	5.0	0.0221	1.6	0.3	137.77	7	141	2	222	158	0.7
TCZ-TA1-27	0.132	12.7	0.0219	1.9	0.2	125.85	15	140	3	61	24	0.4
TCZ-TA1-28	0.148	6.7	0.0224	2.4	0.4	140.49	9	143	3	86	42	0.5
TCZ-TA1-29	0.130	6.3	0.0220	1.9	0.3	123.97	7	140	3	85	81	1.0
**TCZ-TB1**												
TCZ-TB1-1	0.303	4.1	0.0444	2.0	0.5	268.86	10	280	5	71	37	0.5
TCZ-TB1-2	0.136	5.8	0.0220	2.0	0.3	129.15	7	140	3	147	140	1.0
TCZ-TB1-3	0.143	19.5	0.0215	2.5	0.1	135.54	25	137	3	471	77	0.2
TCZ-TB1-4	0.149	4.2	0.0219	1.8	0.4	140.61	6	139	3	207	97	0.5
TCZ-TB1-5	0.275	10.3	0.0369	1.8	0.2	246.88	23	234	4	175	80	0.5
TCZ-TB1-6	0.146	3.7	0.0225	1.7	0.5	138.07	5	143	2	316	298	0.9
TCZ-TB1-7	0.142	5.4	0.0213	1.9	0.4	134.85	7	136	3	207	174	0.8
TCZ-TB1-8	0.160	6.9	0.0228	2.9	0.4	150.42	10	145	4	36	31	0.9
TCZ-TB1-9	0.146	14.3	0.0224	2.6	0.2	138.63	19	143	4	68	29	0.4
TCZ-TB1-10	0.135	5.2	0.0216	2.2	0.4	128.18	6	138	3	131	102	0.8
TCZ-TB1-11	0.139	5.7	0.0221	2.1	0.4	132.51	7	141	3	165	119	0.7
TCZ-TB1-12	0.158	5.0	0.0231	2.2	0.4	149.19	7	147	3	153	86	0.6
TCZ-TB1-13	0.146	4.3	0.0233	1.6	0.4	138.16	6	149	2	627	211	0.3
TCZ-TB1-14	0.151	4.2	0.0225	1.6	0.4	143.12	6	143	2	207	78	0.4
TCZ-TB1-15	0.279	15.8	0.0451	2.4	0.2	250.19	36	284	7	58	17	0.3
TCZ-TB1-16	0.162	5.1	0.0216	3.0	0.6	152.10	7	138	4	74	64	0.9
TCZ-TB1-17	0.152	5.9	0.0216	1.6	0.3	143.47	8	138	2	604	158	0.3
TCZ-TB1-18	0.148	5.0	0.0229	1.8	0.4	140.32	7	146	3	536	513	1.0
TCZ-TB1-19	0.160	6.1	0.0224	1.7	0.3	150.45	9	143	2	487	368	0.8
TCZ-TB1-20	0.141	5.3	0.0224	2.3	0.4	133.92	7	143	3	201	67	0.3
TCZ-TB1-21	0.150	24.9	0.0214	3.2	0.1	141.66	33	137	4	46	55	1.2
TCZ-TB1-22	0.139	5.0	0.0222	2.2	0.4	132.41	6	141	3	109	43	0.4
TCZ-TB1-23	0.145	4.3	0.0216	1.8	0.4	137.36	6	138	2	241	83	0.3
TCZ-TB1-24	0.158	6.6	0.0214	1.8	0.3	148.60	9	136	2	66	41	0.6
TCZ-TB1-25	0.157	5.0	0.0220	1.7	0.3	148.16	7	140	2	343	127	0.4
TCZ-TB1-26	0.139	8.3	0.0221	2.0	0.2	132.42	10	141	3	150	71	0.5

**TJS-TC1**												
TJS-TC1-1	0.144	6.2	0.0236	2.0	0.3	136.83	8	151	3	147	77	0.5
TJS-TC1-2	0.148	21.6	0.0235	2.1	0.1	140.01	29	150	3	219	105	0.5
TJS-TC1-3	0.167	8.6	0.0247	1.9	0.2	156.57	12	158	3	281	185	0.7
TJS-TC1-4	0.157	8.8	0.0242	2.0	0.2	147.82	12	154	3	110	41	0.4
TJS-TC1-5	0.178	4.1	0.0252	1.7	0.4	166.66	6	160	3	205	101	0.5
TJS-TC1-6	0.175	10.5	0.0263	2.2	0.2	163.86	16	167	4	192	95	0.5
TJS-TC1-7	0.161	12.3	0.0260	2.0	0.2	151.57	17	165	3	216	95	0.4
TJS-TC1-8	0.177	5.0	0.0251	2.9	0.6	165.57	8	160	4	129	48	0.4
TJS-TC1-9	0.148	22.9	0.0257	2.8	0.1	139.78	30	164	5	62	26	0.4
TJS-TC1-10	0.323	20.9	0.0439	2.1	0.1	284.20	53	277	6	393	74	0.2
TJS-TC1-11	0.180	22.2	0.0257	2.3	0.1	168.06	35	164	4	155	66	0.4
TJS-TC1-12	0.165	4.4	0.0255	1.7	0.4	155.20	6	162	3	216	90	0.4
TJS-TC1-13	0.167	5.0	0.0254	1.6	0.3	156.61	7	161	3	293	208	0.7
TJS-TC1-14	0.169	5.3	0.0254	2.0	0.4	158.43	8	162	3	174	69	0.4
TJS-TC1-15	0.176	8.0	0.0252	1.9	0.2	164.40	12	161	3	179	77	0.4
TJS-TC1-16	0.173	7.0	0.0253	1.9	0.3	161.75	11	161	3	153	51	0.3
TJS-TC1-17	0.168	4.9	0.0258	2.1	0.4	157.63	7	164	3	288	317	1.1
TJS-TC1-18	0.172	20.5	0.0242	2.1	0.1	161.43	31	154	3	147	103	0.7
TJS-TC1-19	0.175	6.2	0.0250	1.8	0.3	164.06	9	159	3	202	103	0.5
TJS-TC1-20	10.822	2.0	0.4778	1.7	0.9	2507.99	18	2517	35	112	36	0.3
TJS-TC1-21	0.159	9.1	0.0256	2.1	0.2	149.78	13	163	3	216	78	0.4
TJS-TC1-22	0.179	19.7	0.0262	2.3	0.1	166.81	31	167	4	145	90	0.6
TJS-TC1-23	0.154	17.4	0.0250	2.1	0.1	145.60	24	159	3	118	160	1.4
TJS-TC1-24	0.180	4.8	0.0248	1.8	0.4	168.24	7	158	3	285	192	0.7
TJS-TC1-25	0.168	16.4	0.0246	2.1	0.1	157.82	24	157	3	215	183	0.9
TJS-TC1-26	0.167	4.6	0.0249	2.0	0.4	157.00	7	159	3	187	84	0.4
TJS-TC1-27	0.155	4.2	0.0243	1.8	0.4	146.45	6	155	3	435	212	0.5
TJS-TC1-28	0.172	5.7	0.0241	1.8	0.3	160.73	8	153	3	238	117	0.5
TJS-TC1-29	0.173	5.7	0.0236	2.6	0.4	162.42	9	151	4	102	55	0.5
TJS-TC1-30	0.160	13.2	0.0246	3.4	0.3	150.45	19	157	5	86	42	0.5
TJS-TC1-31	10.247	1.6	0.4537	1.5	1.0	2457.34	15	2412	31	231	68	0.3
TJS-TC1-32	0.169	3.8	0.0251	1.8	0.5	158.17	6	160	3	494	630	1.3

TJS-TC1-33	0.174	10.6	0.0254	2.1	0.2	162.60	16	162	3	179	70	0.4
TJS-TC1-34	0.178	15.9	0.0247	2.0	0.1	166.24	25	157	3	186	85	0.5
TJS-TC1-35	0.195	11.7	0.0251	2.3	0.2	181.22	20	160	4	85	40	0.5
TJS-TC1-36	0.160	12.9	0.0252	2.1	0.2	150.32	18	160	3	150	109	0.7
TJS-TC1-37	0.192	9.7	0.0255	2.4	0.2	178.06	16	163	4	152	66	0.4
TJS-TC1-38	0.167	14.0	0.0242	2.8	0.2	156.43	20	154	4	83	35	0.4
TJS-TC1-39	0.169	5.3	0.0254	2.0	0.4	158.43	8	162	3	174	69	0.4
**TCZ-M1**												
TCZ-M1-13	0.149	1.0	0.0236	0.05	0.32	141	8.78	151	3.2	181	103	0.57
TCZ-M1-50	0.177	1.2	0.0238	0.05	0.30	166	10.55	151	3.1	159	71.9	0.45
TCZ-M1-68	0.162	1.5	0.0261	0.06	0.25	152	12.86	166	3.7	97.5	68.0	0.70
TCZ-M1-63	0.274	1.6	0.0389	0.05	0.25	246	12.47	246	3.4	230	181	0.79
TCZ-M1-53	0.270	1.5	0.0390	0.06	0.27	242	11.63	246	3.5	188	191	1.02
TCZ-M1-26	0.280	0.9	0.0390	0.03	0.28	251	7.09	247	2.2	1143	1107	0.97
TCZ-M1-40	0.275	1.3	0.0410	0.05	0.26	247	10.35	247	2.2	264	194	0.73
TCZ-M1-18	0.284	1.0	0.0391	0.04	0.31	254	7.60	247	2.6	633	490	0.77
TCZ-M1-03	0.307	1.2	0.0391	0.04	0.25	272	9.21	247	2.4	475	474	1.00
TCZ-M1-42	0.284	1.5	0.0412	0.06	0.26	254	11.50	249	3.6	267	172	0.65
TCZ-M1-55	0.293	1.8	0.0394	0.06	0.25	261	13.96	249	3.6	136	164	1.21
TCZ-M1-78	0.266	2.4	0.0395	0.11	0.30	239	19.52	250	6.8	41.3	48.8	1.18
TCZ-M1-54	0.276	0.8	0.0396	0.04	0.34	248	6.24	250	2.4	1217	1109	0.91
TCZ-M1-105	0.264	1.1	0.0397	0.05	0.30	238	8.52	251	2.9	374	365	0.97
TCZ-M1-99	0.283	1.5	0.0397	0.06	0.27	253	11.76	251	3.5	216	130	0.60
TCZ-M1-61	0.271	0.9	0.0398	0.04	0.29	244	7.56	252	2.5	537	499	0.93
TCZ-M1-59	0.299	1.2	0.0399	0.05	0.31	265	9.41	252	3.0	262	185	0.70
TCZ-M1-80	0.299	1.1	0.0401	0.05	0.34	265	8.36	254	3.0	583	386	0.66
TCZ-M1-15	0.289	1.2	0.0404	0.04	0.26	258	9.56	255	2.7	359	382	1.07
TCZ-M1-94	0.311	1.0	0.0404	0.04	0.35	275	7.52	256	2.7	950	914	0.96
TCZ-M1-66	0.276	1.6	0.0407	0.06	0.27	248	12.45	257	3.8	164	122	0.74
TCZ-M1-71	0.302	2.6	0.0408	0.09	0.26	268	19.98	258	5.6	73.2	63.6	0.87
TCZ-M1-38	0.277	1.3	0.0410	0.05	0.24	249	10.58	259	2.9	304	245	0.81
TCZ-M1-75	0.296	1.6	0.0414	0.05	0.21	263	12.48	261	2.9	245	191	0.78
TCZ-M1-128	0.315	2.1	0.0419	0.07	0.25	278	16.08	264	4.4	104	74.9	0.72

TCZ-M1-124	0.311	2.2	0.0420	0.07	0.24	275	16.76	265	4.4	85.0	35.2	0.41
TCZ-M1-82	0.328	1.9	0.0433	0.06	0.25	288	14.65	273	3.9	134	220	1.64
TCZ-M1-133	0.335	3.2	0.0436	0.10	0.25	293	24.61	275	6.4	39.3	31.9	0.81
TCZ-M1-136	0.319	2.3	0.0437	0.08	0.27	281	17.41	276	5.2	71.9	66.3	0.92
TCZ-M1-127	0.334	1.8	0.0439	0.07	0.31	293	13.64	277	4.5	123	84.6	0.69
TCZ-M1-131	0.351	2.2	0.0439	0.08	0.27	306	16.57	277	4.6	77.2	84.3	1.09
TCZ-M1-60	0.354	1.6	0.0451	0.05	0.25	308	11.84	284	3.1	207	206	1.00
TCZ-M1-09	0.312	1.9	0.0451	0.07	0.27	276	14.88	284	4.6	145	160	1.11
TCZ-M1-57	0.348	1.8	0.0452	0.07	0.28	303	13.93	285	4.2	141	151	1.07
TCZ-M1-95	0.349	2.0	0.0455	0.07	0.28	304	15.17	287	4.5	106	123	1.16
TCZ-M1-14	0.342	2.3	0.0458	0.09	0.30	299	17.29	289	5.7	71.8	65.8	0.92
TCZ-M1-125	0.359	2.8	0.0459	0.11	0.32	311	20.82	289	7.0	38.2	28.5	0.75
TCZ-M1-132	0.337	1.4	0.0462	0.05	0.27	295	11.01	291	3.4	296	136	0.46
TCZ-M1-27	0.340	1.9	0.0464	0.07	0.26	297	14.71	293	4.2	158	180	1.14
TCZ-M1-37	0.338	1.7	0.0465	0.07	0.28	296	12.88	293	4.0	174	168	0.97
TCZ-M1-05	0.336	1.5	0.0468	0.05	0.25	294	11.55	295	3.2	286	155	0.54
TCZ-M1-23	0.329	1.4	0.0471	0.06	0.30	288	10.46	297	3.7	294	407	1.38
TCZ-M1-64	0.345	3.4	0.0478	0.10	0.22	301	25.63	301	6.4	47.1	36.1	0.77
TCZ-M1-92	0.382	4.6	0.0479	0.19	0.33	328	33.50	302	11.5	25.9	16.8	0.65
TCZ-M1-25	0.353	1.5	0.0480	0.06	0.28	307	11.31	302	3.5	378	132	0.35
TCZ-M1-96	0.367	1.6	0.0514	0.07	0.30	318	11.75	323	4.1	144	69.9	0.48
**TCZ-M2**												
TCZ-M2-40	0.14796	5.92	0.0248	2.20	0.37205	140.1	7.8	158.1	3.4	183	127	0.695
TCZ-M2-38	0.18092	5.94	0.0240	2.42	0.40824	168.9	9.3	153.2	3.7	210	138	0.658
TCZ-M2-37	0.17617	6.19	0.0245	2.41	0.38858	164.8	9.5	156.1	3.7	200	153	0.765
TCZ-M2-36	0.18468	4.16	0.0240	1.96	0.47013	172.1	6.6	152.9	3.0	433	498	1.149
TCZ-M2-35	0.16826	2.57	0.0245	1.60	0.62256	157.9	3.8	155.9	2.5	903	661	0.732
TCZ-M2-34	0.16387	5.64	0.0247	2.09	0.37118	154.1	8.1	157.3	3.3	374	534	1.428
TCZ-M2-33	0.16637	6.05	0.0232	2.25	0.37288	156.3	8.8	147.8	3.3	223	162	0.726
TCZ-M2-32	0.17606	4.16	0.0246	1.92	0.46115	164.7	6.3	156.4	3.0	371	544	1.465
TCZ-M2-31	0.16577	4.94	0.0237	2.03	0.41161	155.7	7.2	150.8	3.0	299	547	1.829
TCZ-M2-30	0.17395	7.01	0.0232	2.60	0.37086	162.8	10.6	148.1	3.8	125	102	0.815
TCZ-M2-29	0.15966	6.55	0.0241	2.17	0.33076	150.4	9.2	153.4	3.3	222	178	0.803

TCZ-M2-28	0.14903	10.05	0.0239	2.18	0.21668	141.1	13.3	152.2	3.3	200	166	0.826
TCZ-M2-27	0.16696	4.24	0.0241	1.92	0.45200	156.8	6.2	153.7	2.9	388	492	1.269
TCZ-M2-26	0.17252	11.14	0.0235	3.51	0.31556	161.6	16.8	149.5	5.2	66	41	0.625
TCZ-M2-25	0.16771	11.27	0.0245	2.01	0.17842	157.4	16.6	156.2	3.1	246	218	0.888
TCZ-M2-24	0.16487	5.12	0.0247	2.13	0.41705	155.0	7.4	157.1	3.3	220	156	0.709
TCZ-M2-23	0.17017	5.09	0.0253	2.18	0.42693	159.6	7.6	161.3	3.5	332	217	0.654
TCZ-M2-22	0.17046	4.44	0.0239	1.97	0.44425	159.8	6.6	152.3	3.0	282	203	0.718
TCZ-M2-21	0.16657	4.94	0.0252	2.06	0.41733	156.4	7.2	160.3	3.3	252	332	1.317
TCZ-M2-20	0.16147	5.86	0.0250	2.18	0.37222	152.0	8.3	159.5	3.4	193	135	0.702
TCZ-M2-19	0.16258	6.42	0.0238	2.36	0.36746	153.0	9.2	151.8	3.5	239	138	0.578
TCZ-M2-17	0.15990	5.08	0.0238	2.23	0.43903	150.6	7.1	151.6	3.3	264	431	1.631
TCZ-M2-16	0.16870	5.21	0.0250	2.14	0.41021	158.3	7.7	159.2	3.4	212	163	0.769
TCZ-M2-15	0.15499	4.85	0.0244	1.94	0.39942	146.3	6.6	155.7	3.0	328	290	0.884
TCZ-M2-14	0.16939	10.90	0.0243	5.02	0.46061	158.9	16.2	154.9	7.7	53	38	0.704
TCZ-M2-13	0.16275	3.55	0.0242	1.76	0.49714	153.1	5.1	154.2	2.7	503	807	1.606
TCZ-M2-12	0.15087	15.06	0.0259	2.83	0.18768	142.7	20.2	164.9	4.6	96	103	1.067
TCZ-M2-11	0.16119	9.98	0.0244	1.98	0.19833	151.7	14.2	155.4	3.0	282	193	0.687
TCZ-M2-10	0.15706	4.47	0.0242	1.95	0.43590	148.1	6.2	154.1	3.0	316	330	1.044
TCZ-M2-1	0.16173	5.11	0.0235	2.01	0.39429	152.2	7.2	149.9	3.0	269	226	0.839
TCZ-M2-9	0.15139	6.58	0.0238	2.08	0.31633	143.1	8.8	151.8	3.1	252	213	0.845
TCZ-M2-8	0.15979	4.86	0.0246	2.00	0.41063	150.5	6.8	156.7	3.1	279	202	0.725
TCZ-M2-7	0.14985	5.61	0.0243	2.18	0.38874	141.8	7.5	155.0	3.3	207	163	0.786
TCZ-M2-6	0.17392	5.24	0.0246	2.29	0.43635	162.8	7.9	156.9	3.5	263	217	0.826
TCZ-M2-5	0.15648	7.47	0.0239	2.17	0.29103	147.6	10.3	152.6	3.3	204	145	0.707
TCZ-M2-4	0.19647	8.95	0.0242	3.37	0.37676	182.1	15.0	154.4	5.1	75	89	1.189
TCZ-M2-3	0.16158	4.04	0.0243	1.86	0.46079	152.1	5.7	154.6	2.8	386	495	1.282
TCZ-M2-2	0.14945	5.02	0.0242	2.15	0.42807	141.4	6.6	154.1	3.3	259	224	0.866

Supplementary Table 3.

Ages of the Tiaojishan and Tuchengzi Formations from the Chicheng, Luanping and Chengde basins in the north margin of the North China Craton.

Basin	Formation	Lithology	Age (Ma)	Method	Reference
Chicheng	Top of Tuchengzi	volcanic rock	141.8 ± 1.1	U–Pb (SIMS)	This study (TCZ-TA1)
	Top of Tuchengzi	volcanic rock	141.1 ± 1.7	U–Pb (SIMS)	This study (TCZ-TB1)
	Middle of Tuchengzi	Sandstone	<151Ma	U–Pb (LA-ICP-MS)	This study (TCZ-M1)
	Middle of Tuchengzi	Andesite	147.5 ± 4.5	U–Pb (SIMS)	This study (TCZ-M2)
	Bottom of Tuchengzi	Sandstone	<149.5	U–Pb (LA-ICP-MS)	Liu et al.[37]
	Top of Tiaojishan	Tuff	153 ± 1.1	U–Pb (SHRIMP)	Xu et al. [14]
	Top of Tiaojishan	Tuff	152.9 ± 2.5	U–Pb (SIMS)	This study (TJS-TC1)
Chengde	Top of Tiaojishan	Tuff (sanidine)	152.6 ± 0.3	^{40}Ar–^{39}Ar	Cope [16]
Luanping	Top of Tiaojishan	Andesite	153.8 ± 5.2	U–Pb (LA-ICP-MS)	Zhang et al. [17]
	Top of Tiaojishan	Andesite	154 ± 4.7	U–Pb (LA-ICP-MS)	Zhang et al. [17]

Supplementary Table 4.

High-temperature component directions of all specimens from the top of Tiaojishan Formation, the middle part of Tuchengzi Formation and the top of Tuchengzi Formation.

Sample ID	D_g	I_g	D_s	I_s	MAD	Strike	Dip	λ_s(°N)	Φ_s(°E)	lithology	Temperature (Range of ChRM)
The top of Tiaojishan Formation (sections TJS-TA, TJS-TB, TJS-TC)											
TJS-TA1	75.1	62.8	27.8	37.4	1.5	263	43	40.83	118.14	volcanic	480~600°C
TJS-TA2	78.2	63.6	27.7	39	2.7	263	43	40.83	118.14	volcanic	480~600°C
TJS-TA3	66.1	66.6	21.1	36.4	2.7	263	43	40.83	118.14	volcanic	500~600°C
TJS-TA4	70.2	64.2	24.9	36.3	1.8	263	43	40.83	118.14	volcanic	500~600°C
TJS-TA5	76.3	67.9	22.3	40.4	1.2	263	43	40.83	118.14	volcanic	500~600°C
TJS-TA6	76	67.2	23	40	2	263	43	40.83	118.14	volcanic	500~600°C
TJS-TA7	74.1	72.8	4.8	45.5	4.4	250	43	40.83	118.14	volcanic	520~600°C
TJS-TA8	87.9	70.1	9.8	49.4	3	250	43	40.83	118.14	volcanic	520~600°C
TJS-TA9	98	68.5	12.5	52.9	6.3	250	43	40.83	118.14	volcanic	520~600°C
TJS-TA10	86	68.1	12.8	48.5	3.5	250	43	40.83	118.14	volcanic	520~600°C
TJS-TA11	80.7	50.6	34.7	40.2	4.8	250	43	40.83	118.14	volcanic	520~600°C
TJS-TA12	92.7	46.4	44.9	45.3	4.1	250	43	40.83	118.14	volcanic	520~600°C
TJS-TA13	91.9	53.1	35.5	47.5	3.3	250	43	40.83	118.14	volcanic	480~600°C
TJS-TA14	112.6	47.6	51.1	58.4	3.2	250	43	40.83	118.14	volcanic	540~600°C
TJS-TA15	113.7	46.9	52.8	58.9	2.4	250	43	40.83	118.14	volcanic	540~600°C
TJS-TA16	118.1	45.2	58	61.2	2.1	250	43	40.83	118.14	volcanic	540~600°C
TJS-TA17	120	43.9	61.4	62.1	4.1	250	43	40.83	118.14	volcanic	540~600°C
TJS-TA18	116.5	45.3	57.2	60.2	2.1	250	43	40.83	118.14	volcanic	520~600°C
TJS-TA19	113.9	51.8	43.5	60.1	4.1	250	43	40.83	118.14	volcanic	500~600°C
TJS-TA20	112.2	51.5	43.7	59.1	2.9	250	43	40.83	118.14	volcanic	500~600°C
TJS-TA21	118.7	45.4	57.8	61.7	1.6	250	43	40.83	118.14	volcanic	500~600°C
TJS-TA22	105.6	57	32.4	56	3.5	250	43	40.83	118.14	volcanic	500~600°C
TJS-TA23	107.4	40	60.9	51.9	3.7	250	43	40.83	118.14	volcanic	500~620°C
TJS-TA24	103.9	43.4	54.1	51.1	1.3	250	43	40.83	118.14	volcanic	500~620°C
TJS-TA25	105.6	43.3	55.1	52.2	1.8	250	43	40.83	118.14	volcanic	500~620°C

TJS-TA26	101.4	49.6	43.7	51.9	2.5	250	43	40.83	118.14	volcanic	500~620°C
TJS-TA28	103.3	42.8	54.6	50.5	1.7	250	43	40.83	118.14	volcanic	500~620°C
TJS-TA29	104.4	41.4	57.2	50.6	1.1	250	43	40.83	118.14	volcanic	500~620°C
TJS-TA30	99.8	42.7	52.9	48.2	1.5	250	43	40.83	118.14	volcanic	500~620°C
TJS-TA31	104.6	41.7	56.9	50.8	1.6	250	43	40.83	118.14	volcanic	500~620°C
TJS-TA32	102.4	47.1	47.9	51.7	1.5	250	43	40.83	118.14	volcanic	500~620°C
TJS-TA33	116.8	49.6	48.7	61.5	4.6	250	43	40.83	118.14	volcanic	540~600°C
TJS-TA34	106.7	47.5	49.1	54.6	4.8	250	43	40.83	118.14	volcanic	540~600°C
TJS-TA35	113.4	56	35	60.3	4.2	250	43	40.83	118.14	volcanic	540~600°C
TJS-TA36	110.6	43.9	56.6	55.8	5.1	250	43	40.83	118.14	volcanic	540~600°C
TJS-TA37	109.1	51.1	43.7	57	5.7	250	43	40.83	118.14	volcanic	540~600°C
TJS-TA38	114.1	47.8	51.3	59.4	7.1	250	43	40.83	118.14	volcanic	560~600°C
TJS-TA39	113.1	38.9	34.3	45.6	1.6	250	70	40.83	118.14	volcanic	600~680°C
TJS-TA40	113	36.7	37.4	45.9	2.5	250	70	40.83	118.14	volcanic	600~680°C
TJS-TA41	111.6	39.8	33.3	44.3	2.5	250	70	40.83	118.14	volcanic	600~680°C
TJS-TA42	114	39.9	32.8	46.1	1.6	250	70	40.83	118.14	volcanic	600~680°C
TJS-TA43	113	40.4	32.2	45.2	1.5	250	70	40.83	118.14	volcanic	600~680°C
TJS-TA44	113.6	38.6	34.6	46.1	1.2	250	70	40.83	118.14	volcanic	600~680°C
TJS-TA45	112.5	39.4	33.7	45	1.4	250	70	40.83	118.14	volcanic	600~680°C
TJS-TA46	108.2	39.5	34.4	41.7	0.6	250	70	40.83	118.14	volcanic	610~680°C
TJS-TA47	117.4	24.7	55.1	50.5	0.5	250	70	40.83	118.14	volcanic	610~680°C
TJS-TA48	121.8	28.9	48	54.2	1.1	250	70	40.83	118.14	volcanic	610~680°C
TJS-TA49	115.1	32.1	43.8	48.2	0.5	250	70	40.83	118.14	volcanic	610~680°C
TJS-TA51	110.1	28.6	49.1	44	1.7	250	70	40.83	118.14	volcanic	610~680°C
TJS-TA52	111.4	38.6	35.1	44.4	0.6	250	70	40.83	118.14	volcanic	610~680°C
TJS-TA53	114.5	33.4	41.9	47.5	0.5	250	70	40.83	118.14	volcanic	610~680°C
TJS-TA54	108.7	38.8	35.2	42.3	0.7	250	70	40.83	118.14	volcanic	610~680°C
TJS-TA55	110	37.2	37.2	43.4	0.9	250	70	40.83	118.14	volcanic	610~680°C
TJS-TA56	126.2	35.5	35.2	56.6	3.4	250	70	40.83	118.14	volcanic	610~680°C
TJS-TA57	122.6	33.9	39.4	54.1	0.8	250	70	40.83	118.14	volcanic	610~680°C
TJS-TA58	122.8	34.7	37.9	54.1	0.9	250	70	40.83	118.14	volcanic	610~680°C
TJS-TA59	127.3	37.5	31.3	56.7	1.1	250	70	40.83	118.14	volcanic	610~680°C

TJS-TA60	123.4	32	42.3	55.2	0.7	250	70	40.83	118.14	volcanic	610~680°C
TJS-TA61	122	32.9	41.1	53.9	0.7	250	70	40.83	118.14	volcanic	610~680°C
TJS-TA62	122.6	34.7	38	54	1.9	250	70	40.83	118.14	volcanic	610~680°C
TJS-TA63	120.8	29.8	46.6	53.3	0.9	250	70	40.83	118.14	volcanic	610~680°C
TJS-TA64	109.9	49.5	21	40.7	1	250	70	40.83	118.14	volcanic	610~680°C
TJS-TA65	108.9	49.1	21.8	40.1	0.9	250	70	40.83	118.14	volcanic	610~680°C
TJS-TA66	104.2	47.9	24.3	37.5	1	250	70	40.83	118.14	volcanic	610~680°C
TJS-TA67	112.3	50	19.8	41.9	1	250	70	40.83	118.14	volcanic	610~680°C
TJS-TA68	118	39.4	32.2	49.2	2	250	70	40.83	118.14	volcanic	610~680°C
TJS-TA70	115.4	36.6	37	47.9	0.7	250	70	40.83	118.14	volcanic	610~680°C
TJS-TA71	120.4	40.9	29.2	50.5	0.8	250	70	40.83	118.14	volcanic	610~680°C
TJS-TA72	113.3	37.8	35.8	46	1.5	250	70	40.83	118.14	volcanic	610~680°C
TJS-TA73	116	39.6	32.6	47.6	2.4	250	70	40.83	118.14	volcanic	610~680°C
TJS-TA74	115.7	39.4	33	47.4	2.8	250	70	40.83	118.14	volcanic	610~680°C
TJS-TA75	112.7	38.7	34.7	45.3	3.1	250	70	40.83	118.14	volcanic	610~680°C
TJS-TA76	105.4	33.1	43.1	40	3.4	250	70	40.83	118.14	volcanic	610~680°C
TJS-TA77	121.5	35.2	37.6	52.9	2.8	250	70	40.83	118.14	volcanic	600~680°C
TJS-TA78	129.7	41.9	22.6	56.3	1.4	250	70	40.83	118.14	volcanic	600~680°C
TJS-TA79	122	35.6	36.8	53.3	0.5	250	70	40.83	118.14	volcanic	600~680°C
TJS-TA80	120.7	35.5	37.4	52.2	1.8	250	70	40.83	118.14	volcanic	600~680°C
TJS-TA81	123	37	34.1	53.7	1.1	250	70	40.83	118.14	volcanic	600~680°C
TJS-TA82	121.5	36.8	35	52.5	0.6	250	70	40.83	118.14	volcanic	600~680°C
TJS-TA83	122.6	36.9	34.5	53.4	1.1	250	70	40.83	118.14	volcanic	600~680°C
TJS-TA84	122.9	36.6	34.9	53.7	1.6	250	70	40.83	118.14	volcanic	600~680°C
TJS-TA85	122.7	34.5	38.3	54.1	1	250	70	40.83	118.14	volcanic	600~680°C
TJS-TA86	118.4	37.7	34.7	49.9	1.8	250	70	40.83	118.14	volcanic	600~680°C
TJS-TA87	115.4	37	36.4	47.7	0.5	250	70	40.83	118.14	volcanic	600~680°C
TJS-TA88	118	37.6	34.9	49.6	0.4	250	70	40.83	118.14	volcanic	600~680°C
TJS-TA89	119.3	38.1	33.8	50.5	2.8	250	70	40.83	118.14	volcanic	600~680°C
TJS-TA90	118.8	35.6	37.8	50.7	0.7	250	70	40.83	118.14	volcanic	600~680°C
TJS-TA91	120	36.3	36.2	51.5	0.4	250	70	40.83	118.14	volcanic	600~680°C
TJS-TA92	118.5	36.6	36.3	50.3	1.2	250	70	40.83	118.14	volcanic	600~680°C

TJS-TA94	117.1	35.9	37.7	49.3	0.8	250	70	40.83	118.14	volcanic	600~680°C
TJS-TA95	118.3	36.4	36.6	50.2	1	250	70	40.83	118.14	volcanic	600~680°C
TJS-TA96	130.9	48.9	12	52.9	4.7	250	70	40.83	118.14	volcanic	600~680°C
TJS-TA97	119.2	35.8	37.4	51	2.9	250	70	40.83	118.14	volcanic	620~680°C
TJS-TA98	124.8	38.3	31.3	54.6	3.2	250	70	40.83	118.14	volcanic	620~680°C
TJS-TA100	125.6	40.6	27.2	54.2	2.7	250	70	40.83	118.14	volcanic	620~680°C
TJS-TB1	351	64.5	348.5	29.5	3.3	256	35	40.8	117.4	volcanic	400~580°C
TJS-TB2	81.5	65.9	25.6	50.4	4.9	256	35	40.8	117.4	volcanic	320~560°C
TJS-TB3	57.4	67	16.5	43	5.3	256	35	40.8	117.4	volcanic	300~520°C
TJS-TB4	38.7	65.4	10.4	36.9	5.2	256	35	40.8	117.4	volcanic	280~540°C
TJS-TB6	42.6	76.6	2.3	46.3	5	256	35	40.8	117.4	volcanic	280~580°C
TJS-TB7	11.9	74.9	354.7	41	4.7	256	35	40.8	117.4	volcanic	280~540°C
TJS-TB8	73.4	63.8	25.7	46.3	5	256	35	40.8	117.4	volcanic	360~580°C
TJS-TB9	48.8	66.3	13.9	40.2	6.3	256	35	40.8	117.4	volcanic	320~580°C
TJS-TB10	29	70.7	2.9	39.4	4.9	256	35	40.8	117.4	volcanic	320~520°C
TJS-TB11	37	64.7	10.2	35.9	6.4	256	35	40.8	117.4	volcanic	320~520°C
TJS-TB12	16.3	68.7	359	35.6	5.3	256	35	40.8	117.4	volcanic	320~540°C
TJS-TB13	18.3	66.6	0.8	34	5	256	35	40.8	117.4	volcanic	280~540°C
TJS-TB15	78.3	64.5	26.5	48.5	5.8	256	35	40.8	117.4	volcanic	520~680°C
TJS-TB16	74.1	62.8	27.2	46.1	5.1	256	35	40.8	117.4	volcanic	520~680°C
TJS-TB17	96.6	60.7	37.9	54.4	4.7	256	35	40.8	117.4	volcanic	650~680°C
TJS-TB18	80.5	61	31.8	47.6	3.5	256	35	40.8	117.4	volcanic	650~680°C
TJS-TB19	79.6	64.7	26.7	49.1	4.9	256	35	40.8	117.4	volcanic	650~680°C
TJS-TB20	86.8	62.3	32.3	50.8	3.5	256	35	40.8	117.4	volcanic	650~680°C
TJS-TB21	79.4	59.3	33.5	46.2	3.9	256	35	40.8	117.4	volcanic	650~680°C
TJS-TB22	92.9	66.6	27.3	54.9	6.4	256	35	40.8	117.4	volcanic	640~680°C
TJS-TB23	36.2	77.1	0.2	45.8	2.9	256	35	40.8	117.4	volcanic	440~560°C
TJS-TB24	27.5	76.2	358.6	43.9	2.4	256	35	40.8	117.4	volcanic	440~560°C
TJS-TB25	56.9	76.6	5.4	48.9	2.6	256	35	40.8	117.4	volcanic	400~560°C
TJS-TB27	17.9	76.5	355.7	43	2.3	256	35	40.8	117.4	volcanic	400~560°C
TJS-TB28	31.8	75.2	0.7	43.6	2.6	256	35	40.8	117.4	volcaniclastic	400~540°C
TJS-TB29	41.2	76.1	2.5	45.8	3.8	256	35	40.8	117.4	volcaniclastic	400~540°C

TJS-TB30	49.4	74.6	6.1	46.2	3.4	256	35	40.8	117.4	volcaniclastic	400~540°C
TJS-TB31	75.3	74.4	11.8	52	2.4	256	35	40.8	117.4	volcaniclastic	400~540°C
TJS-TB32	68.1	70.7	15.5	48.3	4.8	256	35	40.8	117.4	volcaniclastic	400~540°C
TJS-TB34	25.7	64.1	346.2	41.8	2	256	35	40.8	117.4	volcaniclastic	540~670°C
TJS-TB35	25	61.9	348	40.1	2	256	35	40.8	117.4	volcaniclastic	540~680°C
TJS-TB36	26.6	64.5	346.1	42.4	1.7	256	35	40.8	117.4	volcaniclastic	540~680°C
TJS-TB38	27.4	67.2	343.7	44.3	2.8	256	35	40.8	117.4	volcaniclastic	540~680°C
TJS-TB39	24.5	62.8	347	40.5	3.1	256	35	40.8	117.4	volcaniclastic	540~680°C
TJS-TB40	27	64.4	346.4	42.4	2.8	256	35	40.8	117.4	volcaniclastic	540~680°C
TJS-TB44	40.6	64.5	351.3	47.2	2.8	256	35	40.8	117.4	volcaniclastic	540~650°C
TJS-TB45	41.4	64.1	352	47.3	2.7	256	35	40.8	117.4	volcaniclastic	540~650°C
TJS-TC1	245.5	-49.7	220.6	-63.6	4	193	21	40.66	116.18	volcaniclastic	540~610°C
TJS-TC2	243.1	-38.2	227.2	-52.5	12.7	193	21	40.66	116.18	volcaniclastic	600~640°C
TJS-TC5	232.5	-51.4	203.7	-60.6	8.4	193	21	40.66	116.18	volcaniclastic	610~680°C
TJS-TC6	227.3	-33.3	212.4	-42.9	6.2	193	21	40.66	116.18	volcaniclastic	570~680°C
TJS-TC7	240.5	-63	191.4	-72.1	4.6	193	21	40.66	116.18	volcaniclastic	500~580°C
TJS-TC8	221.1	-59.9	181.5	-63.2	9.9	193	21	40.66	116.18	volcaniclastic	580~680°C
TJS-TC12	198.7	-41.7	180.2	-40.4	7.7	193	21	40.66	116.18	volcaniclastic	540~640°C
TJS-TC13	25.8	53.9	356.7	53.2	8.3	193	21	40.66	116.18	volcaniclastic	570~660°C
TJS-TC14	232.8	-59.5	192.3	-67	2.8	193	21	40.66	116.18	volcaniclastic	580~640°C
TJS-TC15	244.8	-39.3	228.4	-54	6	193	21	40.66	116.18	volcaniclastic	650~680°C
TJS-TC18	234.5	-38	217.3	-49.6	7.3	193	21	40.66	116.18	volcaniclastic	640~670°C
TJS-TC19	241.5	-38	225.4	-51.8	9.5	193	21	40.66	116.18	volcaniclastic	630~670°C
TJS-TC20	229.6	-56.1	194.6	-63.4	8.4	193	21	40.66	116.18	volcaniclastic	590~680°C
TJS-TC22	239	-43.3	218.7	-55.9	9.2	193	21	40.66	116.18	volcaniclastic	580~680°C
TJS-TC23	249.4	-31.6	238.2	-48	7.1	193	21	40.66	116.18	volcaniclastic	580~640°C
TJS-TC24	223.5	-31.9	209.2	-40.4	4.2	193	21	40.66	116.18	tuffaceous sandstone	560~670°C
TJS-TC27	27.4	60.8	350.1	59.1	4.9	193	21	40.66	116.18	tuffaceous sandstone	610~660°C
TJS-TC28	12.6	57.8	343	52.1	6.1	193	21	40.66	116.18	tuffaceous sandstone	580~670°C
TJS-TC29	197.2	-38.6	180.7	-37.1	4.4	193	21	40.66	116.18	tuffaceous sandstone	570~680°C
TJS-TC30	43.5	60.8	1.9	64.7	7.2	193	21	40.66	116.18	tuffaceous sandstone	560~680°C
TJS-TC31	242.7	-32.1	230	-46.7	6.5	193	21	40.66	116.18	tuffaceous sandstone	610~680°C

TJS-TC33	4.1	47.3	344.7	40.4	9.1	193	21	40.66	116.18	tuffaceous sandstone	320~520°C
TJS-TC34	15.4	56.8	346.1	52.1	10.2	193	21	40.66	116.18	tuffaceous sandstone	540~600°C
TJS-TC36	246.4	-36.7	232	-52.1	6.6	193	21	40.66	116.18	tuffaceous sandstone	480~590°C
TJS-TC37	50.2	43.9	28.4	53.4	12.6	193	21	40.66	116.18	tuffaceous sandstone	360~500°C
TJS-TC38	224.8	-49.5	197.8	-56.4	10.8	193	21	40.66	116.18	tuffaceous sandstone	560~650°C
TJS-TC44	41.6	65.1	352.6	66.8	6.7	193	21	40.66	116.18	tuffaceous sandstone	440~610°C
TJS-TC45	5.8	55.6	340.2	48.1	3.1	193	21	40.66	116.18	tuffaceous sandstone	570~620°C
TJS-TC46	9.2	39.8	353.1	35.4	3.5	193	21	40.66	116.18	tuffaceous sandstone	540~610°C
TJS-TC49	238.9	-55.8	204.6	-66.5	9.1	193	21	40.66	116.18	tuffaceous sandstone	570~660°C
The middle part of the Tuchengzi Formation (section TCZ-M)											
TCZ-TM1	5.1	23.4	349.7	38	8.9	148	29	40.7	116.1	red sandstone	660~680°C
TCZ-TM2	192.6	-21.1	178.9	-39.2	10.2	148	29	40.7	116.1	red sandstone	620~660°C
TCZ-TM3	15	-16.5	17	5.2	8	148	29	40.7	116.1	red sandstone	640~680°C
TCZ-TM4	24.1	34.3	3.3	55.6	8.3	148	29	40.7	116.1	red sandstone	650~680°C
TCZ-TM5	13.9	1.3	9.6	21.6	6.3	148	29	40.7	116.1	red sandstone	600~680°C
TCZ-TM6	349.2	-0.8	347.1	9.4	4.6	148	29	40.7	116.1	red sandstone	600~650°C
TCZ-TM9	7.7	30.5	347.5	45.3	9.7	148	29	40.7	116.1	red sandstone	630~660°C
TCZ-TM10	7.5	6.4	1	23.8	2.2	148	29	40.7	116.1	red sandstone	630~660°C
TCZ-TM11	3.3	25.8	346.3	39.3	4	148	29	40.7	116.1	red sandstone	630~660°C
TCZ-TM12	33.1	25.2	21.3	50.4	5.1	148	29	40.7	116.1	red sandstone	630~660°C
TCZ-TM13	14.5	-0.9	11	19.8	3.8	148	29	40.7	116.1	red sandstone	650~680°C
TCZ-TM14	14.6	9.1	7	29.1	2	148	29	40.7	116.1	red sandstone	630~680°C
TCZ-TM15	11.1	2	6.5	21.2	2.5	148	29	40.7	116.1	red sandstone	630~680°C
TCZ-TM16	1.2	19.1	348.4	32.5	1.2	148	29	40.7	116.1	red sandstone	630~670°C
TCZ-TM17	23.6	18.3	12.8	40.8	1.2	148	29	40.7	116.1	red sandstone	630~670°C
TCZ-TM18	25.2	8.6	18.8	32.3	10	148	29	40.7	116.1	red sandstone	630~680°C
TCZ-TM19	26.1	8	19.9	32	3.6	148	29	40.7	116.1	red sandstone	660~680°C
TCZ-TM20	3.9	-1.9	1.1	14.8	8.2	148	29	40.7	116.1	red sandstone	630~680°C
TCZ-TM22	2	-4.4	0.5	11.7	11.4	148	29	40.7	116.1	red sandstone	620~660°C
TCZ-TM23	23.7	1.4	19.6	25	4.7	148	29	40.7	116.1	red sandstone	630~680°C
TCZ-TM24	24	-3	21.3	20.8	6.3	148	29	40.7	116.1	red sandstone	630~680°C
TCZ-TM25	356.8	8.2	349.9	20.8	7.8	148	29	40.7	116.1	red sandstone	585~650°C

TCZ-TM26	17.6	1.3	13.3	22.8	9	148	29	40.7	116.1	red sandstone	660~680°C
TCZ-TM27	347	7.7	341	15.9	5.5	148	29	40.7	116.1	red sandstone	620~660°C
TCZ-TM28	14.7	17.6	3.1	36.9	12.9	148	29	40.7	116.1	red sandstone	640~680°C
TCZ-TM29	17	10.1	9.2	30.9	6.3	148	29	40.7	116.1	red sandstone	620~680°C
TCZ-TM30	16	27.1	359	46	7.9	148	29	40.7	116.1	red sandstone	620~670°C
TCZ-TM32	36.7	34.1	21.3	59.8	11.9	148	29	40.7	116.1	red sandstone	620~670°C
TCZ-TM33	1.6	15	351	29	1.5	148	29	40.7	116.1	red sandstone	620~670°C
TCZ-TM34	13.1	25	357.1	42.9	3.4	148	29	40.7	116.1	red sandstone	620~680°C
TCZ-TM35	31.4	-1.8	28.7	23.9	9.3	148	29	40.7	116.1	red sandstone	620~680°C
TCZ-TM36	32.9	3.2	28.9	29.2	12.5	148	29	40.7	116.1	red sandstone	620~680°C
TCZ-TM37	42.3	1.7	39.9	29.5	4.2	148	29	40.7	116.1	red sandstone	620~670°C
TCZ-TM38	23.7	-7.1	22.2	16.8	8.8	148	29	40.7	116.1	red sandstone	630~660°C
TCZ-TM40	353.1	6.3	347.3	17.4	7.6	148	29	40.7	116.1	red sandstone	640~680°C
TCZ-TM41	27.9	-0.6	24.7	24.2	15	148	29	40.7	116.1	red sandstone	600~680°C
TCZ-TM42	40.4	19.4	33.5	46.6	5.7	148	29	40.7	116.1	red sandstone	600~680°C
TCZ-TM44	5.9	-3.9	3.8	13.8	7.1	148	29	40.7	116.1	red sandstone	600~680°C
TCZ-TM45	25	-2.5	22.2	21.6	6.7	148	29	40.7	116.1	red sandstone	630~680°C
TCZ-TM46	9.2	-3	6.6	15.8	9.3	148	29	40.7	116.1	red sandstone	620~680°C
TCZ-TM47	10.8	10.2	2.7	28.6	14.7	148	29	40.7	116.1	red sandstone	600~680°C
TCZ-TM48	28.2	10.7	21.3	35.1	9	148	29	40.7	116.1	red sandstone	610~680°C
TCZ-TM50	216.8	-19	209	-45.3	6.6	148	29	40.7	116.1	red sandstone	620~660°C
TCZ-TM51	33.6	19.5	24.5	45.1	2.8	148	29	40.7	116.1	red sandstone	620~670°C
TCZ-TM52	34.8	10.8	29	37	3.7	148	29	40.7	116.1	red sandstone	600~670°C
TCZ-TM53	40.9	16.1	35.1	43.5	6.9	148	29	40.7	116.1	red sandstone	650~680°C
TCZ-TM54	40.7	16.2	34.8	43.5	13.7	148	29	40.7	116.1	red sandstone	600~670°C
TCZ-TM55	45.5	6	42.9	34.1	8.8	148	29	40.7	116.1	red sandstone	660~680°C
TCZ-TM58	46.6	14.5	42.9	42.8	7.9	148	29	40.7	116.1	red sandstone	640~680°C
TCZ-TM59	2.7	18.7	350.2	32.8	8.6	148	29	40.7	116.1	red sandstone	660~680°C
TCZ-TM60	13.2	34.9	349.8	51.5	0	148	29	40.7	116.1	red sandstone	640~660°C
TCZ-TM61	53.2	42.5	47	71.2	9	148	29	40.7	116.1	red sandstone	600~650°C
TCZ-TM62	20.2	33.6	359	53.4	8.2	148	29	40.7	116.1	red sandstone	630~650°C
TCZ-TM65	44.7	15.1	40.2	43.1	3.2	148	29	40.7	116.1	red sandstone	620~650°C

TCZ-TM66	16	7.8	9.1	28.4	6.2	148	29	40.7	116.1	red sandstone	620~650°C
TCZ-TM67	2.2	30.2	342.3	42.5	6.9	148	29	40.7	116.1	red sandstone	640~670°C
TCZ-TM68	38.3	23.7	29	50.3	12.4	148	29	40.7	116.1	red sandstone	650~680°C
TCZ-TM70	9.4	45	335.1	57.7	4.5	148	29	40.7	116.1	red sandstone	640~680°C
TCZ-TM71	35.4	42.7	11.1	67.2	10.6	148	29	40.7	116.1	red sandstone	650~680°C
TCZ-TM72	19.2	30.8	0.1	50.6	3.4	148	29	40.7	116.1	red sandstone	630~650°C
TCZ-TM73	27.7	33.1	9.1	55.9	13.6	148	29	40.7	116.1	red sandstone	630~670°C
TCZ-TM76	18.1	29.4	359.8	48.9	3.1	148	29	40.7	116.1	red sandstone	630~670°C
TCZ-TM77	32.3	43.3	5.4	66.6	5.4	148	29	40.7	116.1	red sandstone	640~670°C
TCZ-TM78	13.3	45.5	338.4	59.9	1.5	148	29	40.7	116.1	red sandstone	640~670°C
TCZ-TM79	11.7	23.2	356.7	40.7	7.7	148	29	40.7	116.1	red sandstone	630~660°C
TCZ-TM80	13.4	36.9	348.3	53.2	3.3	148	29	40.7	116.1	red sandstone	650~680°C
TCZ-TM81	27.1	42.3	359.1	63.7	2.9	148	29	40.7	116.1	red sandstone	650~680°C
TCZ-TM85	16.4	39.9	348.6	57	7	148	29	40.7	116.1	red sandstone	650~680°C
TCZ-TM86	15.8	33.2	354.2	51.2	4.1	148	29	40.7	116.1	red sandstone	650~680°C
TCZ-TM87	15.5	24.1	0.4	43	3.3	148	29	40.7	116.1	red sandstone	650~680°C
TCZ-TM88	18.6	31.7	358.7	51.1	2.2	148	29	40.7	116.1	red sandstone	650~680°C
TCZ-TM89	18.6	24	3.9	44.3	4.6	148	29	40.7	116.1	red sandstone	650~680°C
TCZ-TM90	22.2	21.2	9.7	43.1	8.2	148	29	40.7	116.1	red sandstone	650~680°C
TCZ-TM94	204.1	10.7	203.7	-13.5	4.9	148	29	40.7	116.1	red sandstone	650~680°C
TCZ-TM96	2.5	-3.9	0.6	12.4	6.5	148	29	40.7	116.1	red sandstone	600~660°C
TCZ-TM97	16.6	15.7	6.1	36	10.2	148	29	40.7	116.1	red sandstone	600~650°C
TCZ-TM100	357.7	-1.6	355.3	12.4	5.7	148	29	40.7	116.1	red sandstone	650~680°C
TCZ-TM102	0.4	1	356.6	16	14.7	148	29	40.7	116.1	red sandstone	630~670°C
TCZ-TM105	344.8	-4.4	344.9	4.2	11	148	29	40.7	116.1	red sandstone	540~670°C
TCZ-TM106	351.1	3.6	346.7	14.2	5.8	148	29	40.7	116.1	red sandstone	650~680°C
TCZ-TM107	229	-3.4	227.4	-32	8.8	148	29	40.7	116.1	red sandstone	650~680°C
TCZ-TM108	208.8	-25.1	195.7	-48.9	12.2	148	29	40.7	116.1	red sandstone	640~680°C
TCZ-TM109	203.3	-11	195.7	-33.9	8.5	148	29	40.7	116.1	red sandstone	650~680°C
TCZ-TM112	205.1	-31	187.2	-53	5.5	148	29	40.7	116.1	red sandstone	650~680°C
TCZ-TM113	216.9	-22	207.9	-48.4	4.3	148	29	40.7	116.1	red sandstone	650~680°C
TCZ-TM114	223.3	-15.2	218.5	-43	7.6	148	29	40.7	116.1	red sandstone	650~680°C

TCZ-TM115	187.5	4.9	185.8	-13.5	13.7	148	29	40.7	116.1	red sandstone	650~680°C
TCZ-TM118	19	33.1	358.1	52.5	12.4	148	29	40.7	116.1	red sandstone	610~680°C
TCZ-TM119	217.5	-30.5	204.7	-56.6	13.7	148	29	40.7	116.1	red sandstone	620~680°C
TCZ-TM120	23	24.4	8.9	46.3	13.8	148	29	40.7	116.1	red sandstone	640~670°C
TCZ-M121	5.7	-5.6	4.4	12.1	10.8	148	29	40.7	116.1	red sandstone	540~580°C
TCZ-M122	27.9	6.5	22.5	31.1	10.8	148	29	40.7	116.1	red sandstone	610~680°C
TCZ-M123	30	1.3	26.3	26.6	7.3	148	29	40.7	116.1	red sandstone	620~680°C
TCZ-M124	31.2	9.4	25.2	34.8	7.1	148	29	40.7	116.1	red sandstone	600~680°C
TCZ-M125	24.8	11.5	17.2	34.9	13.2	148	29	40.7	116.1	red sandstone	580~680°C
TCZ-M126	23	44.5	350.8	63.6	5.9	148	29	40.7	116.1	red sandstone	450~600°C
TCZ-M127	18.3	49.7	337	65.3	8.7	148	29	40.7	116.1	red sandstone	450~600°C
TCZ-M128	24.1	43.8	353.1	63.6	6.7	148	29	40.7	116.1	red sandstone	450~600°C
TCZ-M129	38.5	8.3	34.1	35.4	8.4	148	29	40.7	116.1	red sandstone	600~680°C
TCZ-M130	24.3	28.2	8.1	50.3	7.8	148	29	40.7	116.1	red sandstone	560~610°C
TCZ-M131	34.6	23.9	24	49.6	10.6	148	29	40.7	116.1	red sandstone	540~600°C
TCZ-M132	30.7	18.1	21.4	43	10.3	148	29	40.7	116.1	red sandstone	540~600°C
TCZ-M133	18.1	33.7	356.5	52.6	14.3	148	29	40.7	116.1	red sandstone	540~610°C
TCZ-M134	316.9	66.1	275.8	49.6	8.5	148	29	40.7	116.1	Pyroclastic rock	450~580°C
TCZ-M135	303.7	55.9	278.2	37.8	2.8	148	29	40.7	116.1	Pyroclastic rock	400~580°C
TCZ-M136	296.4	-41.6	326.2	-50.4	7.1	148	29	40.7	116.1	Pyroclastic rock	450~580°C
TCZ-M137	329.1	27.1	314.9	24	12.7	148	29	40.7	116.1	Pyroclastic rock	520~580°C
TCZ-M138	244.4	31.7	243.5	2.8	4.6	148	29	40.7	116.1	Pyroclastic rock	450~580°C
TCZ-M139	331.4	58.4	291.6	49.5	5.7	148	29	40.7	116.1	Pyroclastic rock	400~580°C
TCZ-M140	263.4	-52.1	306.8	-73.6	1.3	148	29	40.7	116.1	Pyroclastic rock	600~680°C
TCZ-M141	267.3	-55.6	321.6	-73.8	3	148	29	40.7	116.1	Pyroclastic rock	610~680°C
TCZ-M142	342.8	46.4	311.5	46	6.5	148	29	40.7	116.1	Pyroclastic rock	450~580°C
TCZ-M143	324.2	58.2	287.9	46.6	8.4	148	29	40.7	116.1	pyroclastic rock	500~580°C
The top of Tuchengzi Formation (sections TCZ-TA and TCZ-TB)											
TCZ-TA1	0.6	50.5	1.2	30.5	2.6	273	20	40.71972	115.8667	tuffaceous sandstone	540~620°C
TCZ-TA2	331.3	62.2	343	44.1	3.9	273	20	40.71972	115.8667	tuffaceous sandstone	540~620°C
TCZ-TA3	25.6	65.2	16.4	46.1	4.4	273	20	40.71972	115.8667	tuffaceous sandstone	540~620°C
TCZ-TA4	85.8	68.4	48.4	59.1	3.4	273	20	40.71972	115.8667	tuffaceous sandstone	540~620°C

TCZ-TA5	67.7	71.2	35.7	57.4	8.6	273	20	40.71972	115.8667	tuffaceous sandstone	540~600°C
TCZ-TA7	7.7	77.6	4.8	57.6	1.4	273	20	40.71972	115.8667	volcaniclastic	520~600°C
TCZ-TA8	13.8	74.5	7.9	54.7	2.7	273	20	40.71972	115.8667	volcaniclastic	540~600°C
TCZ-TA9	342.8	77.7	355	58.2	1.7	273	20	40.71972	115.8667	volcaniclastic	540~600°C
TCZ-TA11	28.5	78.7	12.5	59.4	1.8	273	20	40.71972	115.8667	volcaniclastic	540~600°C
TCZ-TA12	347	76.6	356.3	56.9	2.3	273	20	40.71972	115.8667	volcaniclastic	540~620°C
TCZ-TA13	42.9	71.1	23.4	53.5	2.8	273	20	40.71972	115.8667	volcaniclastic	540~620°C
TCZ-TA14	13.5	72.1	8.3	52.2	3	273	20	40.71972	115.8667	volcaniclastic	540~620°C
TCZ-TA15	38.8	75.7	18.5	57.4	3.7	273	20	40.71972	115.8667	volcaniclastic	540~600°C
TCZ-TA17	351.5	64.4	356	44.6	3.6	273	20	40.71972	115.8667	volcaniclastic	540~620°C
TCZ-TA18	349.8	52.5	353.5	32.9	7.3	273	20	40.71972	115.8667	volcaniclastic	400~580°C
TCZ-TA19	285.5	78.1	335	64.7	5.6	273	20	40.71972	115.8667	volcaniclastic	430~620°C
TCZ-TA20	62.9	80.4	22.1	63.9	5.3	273	20	40.71972	115.8667	tuffaceous sandstone	430~520°C
TCZ-TA23	38.2	70	21.6	51.9	4.1	273	20	40.71972	115.8667	tuffaceous sandstone	540~600°C
TCZ-TA24	356.6	52.3	358.4	32.4	7.2	273	20	40.71972	115.8667	tuffaceous sandstone	540~620°C
TCZ-TA25	337.1	76.5	352.1	57.3	9.5	273	20	40.71972	115.8667	tuffaceous sandstone	540~600°C
TCZ-TA26	12.6	67.5	8.4	47.7	9.1	273	20	40.71972	115.8667	tuffaceous sandstone	560~620°C
TCZ-TA27	60.2	69.6	33.4	54.7	4.8	273	20	40.71972	115.8667	tuffaceous sandstone	540~600°C
TCZ-TA29	0.3	75.1	1.8	55.1	4.6	273	20	40.71972	115.8667	tuffaceous sandstone	560~600°C
TCZ-TA30	35.5	69.6	20.4	51.3	8.5	273	20	40.71972	115.8667	tuffaceous sandstone	560~600°C
TCZ-TA31	303.1	86	353.9	67.7	3.4	273	20	40.71972	115.8667	tuffaceous sandstone	540~600°C
TCZ-TA32	8.7	70.3	6	50.4	4	273	20	40.71972	115.8667	tuffaceous sandstone	520~600°C
TCZ-TA33	349.5	70	355.8	50.3	5.3	273	20	40.71972	115.8667	volcaniclastic	540~600°C
TCZ-TA35	212.2	-70.2	198.4	-51.5	3.8	273	20	40.71972	115.8667	volcaniclastic	540~600°C
TCZ-TA36	347.3	64.1	353.5	44.5	7.3	273	20	40.71972	115.8667	volcaniclastic	520~600°C
TCZ-TA37	68.9	86	12.9	68.1	11.2	273	20	40.71972	115.8667	volcaniclastic	540~600°C
TCZ-TA38	19.3	70.9	11.5	51.3	6.1	273	20	40.71972	115.8667	tuffaceous sandstone	560~660°C
TCZ-TA39	59.4	72.4	30.5	57	9.8	273	20	40.71972	115.8667	tuffaceous sandstone	580~640°C
TCZ-TA45	81.9	83.4	20.3	67.8	0.8	273	20	40.71972	115.8667	tuffaceous sandstone	560~600°C
TCZ-TA46	9.1	64.1	6.7	44.1	5.8	273	20	40.71972	115.8667	tuffaceous sandstone	540~620°C
TCZ-TA47	25.4	72	14.2	52.8	10.2	273	20	40.71972	115.8667	tuffaceous sandstone	540~600°C
TCZ-TA48	23.6	71.7	13.5	52.4	3	273	20	40.71972	115.8667	tuffaceous sandstone	490~580°C

TCZ-TA51	212.3	-62.2	201.4	-43.8	8.7	273	20	40.71972	115.8667	tuffaceous sandstone	490~580°C
TCZ-TA52	163.3	-63.2	170.8	-43.9	4.8	273	20	40.71972	115.8667	tuffaceous sandstone	640~690°C
TCZ-TA53	164.4	-61.5	171.2	-42.2	1	273	20	40.71972	115.8667	tuffaceous sandstone	540~600°C
TCZ-TA54	177.8	-59.8	179.6	-39.9	1.4	273	20	40.71972	115.8667	tuffaceous sandstone	660~690°C
TCZ-TA57	148.8	-64.2	162.2	-46.3	2.5	273	20	40.71972	115.8667	tuffaceous sandstone	520~620°C
TCZ-TB59	326.6	63	26.6	70.7	7.3	12	25	40.71972	115.8167	tuffaceous sandstone	600~640°C
TCZ-TB60	355.9	61.2	40.2	58.2	8.6	12	25	40.71972	115.8167	tuffaceous sandstone	630~670°C
TCZ-TB61	348.2	69.8	52.5	65.5	10.6	12	25	40.71972	115.8167	tuffaceous sandstone	580~620°C
TCZ-TB62	45.6	67.1	73	48.1	5.9	12	25	40.71972	115.8167	tuffaceous sandstone	560~620°C
TCZ-TB65	31.6	53	54.5	39.7	8.9	12	25	40.71972	115.8167	tuffaceous sandstone	560~620°C
TCZ-TB66	53.1	55.1	69.9	35.8	7.4	12	25	40.71972	115.8167	tuffaceous sandstone	400~620°C
TCZ-TB67	42.1	60.5	66.2	43.2	8.1	12	25	40.71972	115.8167	tuffaceous sandstone	520~620°C
TCZ-TB70	11.4	68.1	58.2	57.5	6.4	12	25	40.71972	115.8167	tuffaceous sandstone	440~580°C
TCZ-TB71	329.7	61.9	25.8	69	4.1	12	25	40.71972	115.8167	tuffaceous sandstone	480~620°C
TCZ-TB72	67.4	73.7	87.5	50.6	3.1	12	25	40.71972	115.8167	tuffaceous sandstone	400~620°C
TCZ-TB73	62.2	63.5	79.4	41.8	6.4	12	25	40.71972	115.8167	tuffaceous sandstone	520~620°C
TCZ-TB75	30.1	60.1	58.9	46.1	8.3	12	25	40.71972	115.8167	tuffaceous sandstone	400~600°C
TCZ-TB76	326.8	59.8	18.8	69.1	5.6	12	25	40.71972	115.8167	tuffaceous sandstone	520~620°C
TCZ-TB77	350.9	60.3	35.9	59.6	8.8	12	25	40.71972	115.8167	tuffaceous sandstone	520~620°C
TCZ-TB78	1.6	46.7	28.2	45.4	5	12	25	40.71972	115.8167	tuffaceous sandstone	540~620°C
TCZ-TB85	62.9	43.4	72.2	22.6	4.6	12	25	40.71972	115.8167	tuffaceous sandstone	500~580°C
TCZ-TB86	334.1	78.6	74.8	70	5.7	12	25	40.71972	115.8167	tuffaceous sandstone	440~680°C
TCZ-TB87	345.9	57.9	29	60.1	2.8	12	25	40.71972	115.8167	tuffaceous sandstone	600~680°C
TCZ-TB90	348.3	49.2	19.8	52.9	2.1	12	25	40.71972	115.8167	tuffaceous sandstone	600~680°C
TCZ-TB91	333.7	59.7	23.7	66.2	3.1	12	25	40.71972	115.8167	tuffaceous sandstone	540~630°C
TCZ-TB92	357.3	43.1	21.8	44.2	5.5	12	25	40.71972	115.8167	tuffaceous sandstone	480~580°C
TCZ-TB93	42.7	68.1	72.4	49.5	2.6	12	25	40.71972	115.8167	tuffaceous sandstone	540~680°C
TCZ-TB94	43.8	54.8	63.7	37.7	1	12	25	40.71972	115.8167	tuffaceous sandstone	540~680°C
TCZ-TB95	17.2	53.1	44.9	44.6	6	12	25	40.71972	115.8167	tuffaceous sandstone	440~620°C
TCZ-TB97	14.7	50.5	41	43.4	4	12	25	40.71972	115.8167	tuffaceous sandstone	520~620°C
TCZ-TB98	57.5	50.1	70.7	30.1	3.5	12	25	40.71972	115.8167	tuffaceous sandstone	400~580°C
TCZ-TB99	351.6	57.5	32.4	57.5	10.5	12	25	40.71972	115.8167	tuffaceous sandstone	520~600°C

TCZ-TB100	12.6	36.9	30.1	32.8	2.4	12	25	40.71972	115.8167	tuffaceous sandstone	540~620°C
TCZ-TB101	4.9	55.4	38.8	50.8	3.6	12	25	40.71972	115.8167	tuffaceous sandstone	540~640°C
TCZ-TB103	308.5	69.2	46	79	11.7	12	25	40.71972	115.8167	tuffaceous sandstone	600~640°C
TCZ-TB104	12.2	55.2	43.4	48	3.4	12	25	40.71972	115.8167	tuffaceous sandstone	560~640°C
TCZ-TB105	34.5	52.7	56.2	38.5	3.3	12	25	40.71972	115.8167	tuffaceous sandstone	560~640°C
TCZ-TB107	344.4	75.4	65.5	67.8	2.8	12	25	40.71972	115.8167	tuffaceous sandstone	540~620°C
TCZ-TB108	8.6	64.2	51.5	55.8	1.6	12	25	40.71972	115.8167	tuffaceous sandstone	540~640°C
TCZ-TB109	44	50.4	61.4	33.8	4.8	12	25	40.71972	115.8167	tuffaceous sandstone	540~640°C
TCZ-TB115	32.2	74	74.2	56.1	3.5	12	25	40.71972	115.8167	tuffaceous sandstone	540~620°C
TCZ-TB116	28.2	55.7	54.3	43	4.6	12	25	40.71972	115.8167	tuffaceous sandstone	540~640°C
TCZ-TB117	37.3	76.6	79.3	57.1	2.9	12	25	40.71972	115.8167	tuffaceous sandstone	480~580°C
TCZ-TB118	20.2	54.8	48.4	44.9	6.9	12	25	40.71972	115.8167	tuffaceous sandstone	600~680°C
TCZ-TB121	7.3	41	28.3	38.4	9.9	12	25	40.71972	115.8167	tuffaceous sandstone	600~660°C
TCZ-TB122	40.6	56.4	62.6	40	11.4	12	25	40.71972	115.8167	tuffaceous sandstone	580~630°C
TCZ-TB123	339.6	57	23	62.1	6.1	12	25	40.71972	115.8167	tuffaceous sandstone	520~660°C
TCZ-TB124	14.3	66.5	57.3	55.5	7.8	12	25	40.71972	115.8167	tuffaceous sandstone	600~680°C
TCZ-TB125	49	76.1	82.5	54.9	10.4	12	25	40.71972	115.8167	tuffaceous sandstone	620~680°C
TCZ-TB126	39	61.7	65.3	45	7.4	12	25	40.71972	115.8167	tuffaceous sandstone	540~620°C
TCZ-TB130	335.1	59.5	24.3	65.5	12.5	12	25	40.71972	115.8167	tuffaceous sandstone	580~680°C
TCZ-TB131	16	54.5	45.3	46.1	4.1	12	25	40.71972	115.8167	tuffaceous sandstone	560~620°C
TCZ-TB132	356.4	66.6	49.2	61.3	3.8	12	25	40.71972	115.8167	tuffaceous sandstone	440~600°C
TCZ-TB133	355.2	70.5	56.2	63.5	4.7	12	25	40.71972	115.8167	volcaniclastic	520~680°C
TCZ-TB134	42.4	64.5	69.3	46.5	7.7	12	25	40.71972	115.8167	volcaniclastic	520~680°C
TCZ-TB136	29.2	47	49	35.3	8.4	12	25	40.71972	115.8167	volcaniclastic	500~600°C
TCZ-TB137	350.6	69.9	53.4	64.8	2.2	12	25	40.71972	115.8167	volcaniclastic	500~600°C
TCZ-TB138	39.1	62.1	65.7	45.3	3.5	12	25	40.71972	115.8167	volcaniclastic	540~680°C
TCZ-TB139	360	59.8	40.8	55.8	2.2	12	25	40.71972	115.8167	volcaniclastic	520~630°C
TCZ-TB140	15	46	38	39.5	6.6	12	25	40.71972	115.8167	volcaniclastic	600~680°C
TCZ-TB141	8.2	68.3	57.2	58.5	5	12	25	40.71972	115.8167	volcaniclastic	500~600°C
TCZ-TB142	336.3	42.1	1.7	52.2	8.4	12	25	40.71972	115.8167	volcaniclastic	640~670°C
TCZ-TB143	24.3	82.6	86.1	62.5	1	12	25	40.71972	115.8167	volcaniclastic	500~650°C
TCZ-TB144	57.4	62.5	76.3	41.7	10.8	12	25	40.71972	115.8167	volcaniclastic	640~680°C

TCZ-TB145	38.2	67	69.3	49.6	4.3	12	25	40.71972	115.8167	volcaniclastic	500~680°C
TCZ-TB146	48.6	58.2	68.7	39.6	3	12	25	40.71972	115.8167	volcaniclastic	500~680°C
TCZ-TB147	27.6	68.6	65.9	53.3	3.1	12	25	40.71972	115.8167	volcaniclastic	500~680°C
TCZ-TB148	65.1	65.6	82	43.3	6.4	12	25	40.71972	115.8167	volcaniclastic	440~600°C

Abbreviations: Strike/dip, strike azimuth and dip of bed; D_g, I_g (D_s, I_s), declination and inclination of direction in situ (tilt adjusted); a_{95}, precision parameter and the radius of the cone of 95% confidence for direction. λ_s, φ_s, latitude and longitude of sampling site.

.

Supplementary Table 5.

Late Triassic to Cretaceous paleomagnetic poles from the North China Craton.

NO.	Age (Ma)	Locality	Rock type	Slat. (°N)	Slong. (°E)	N(n)	Plat. (°N)	Plong. (°E)	A_{95}(°) (dp/dm)	Paleolatitude (°N)	Test	References
A	179	Datong, Shanxi	sandstone	40	113.1	5S	81.5	302.5	5.5	32.5	F	Uno & Huang [38]
B	170	Beipiao, Liaoning	volcanic rocks	41.2	120.7	20S	89.3	216.8	5.4/7.1	40.9	B	Gao et al. [39]
C	165	Beipiao, Liaoning	volcanic rocks	41.1	120.6	9S	84.1	228.9	6.6	39.0	B	Gao et al. [39]
D	160	Beipiao, Liaoning	volcanic rocks	41.3	120.7	27S	82.7	282.6	3.2	34	F&B	Gao et al. [39]
E	155	Hebei & Liaoning	volcanic rocks	40.60 ~ 41.81	117.44 ~ 120.60	51S	66.1	208.3	5.1	37.9	F&R	Yi et al. [40]
F	153	Zhangjiakou & Chengde & Luanping, Hebei	volcanic & pyroclastic rocks	40.66 ~ 40.83	116.18 ~ 118.14	162s	77.3	249.1	2.1	32.5	F	This study
G	~147*	Zhangjiakou, Hebei	sandstone	40.7	116.1	103s	72.3	268.2	3.9	25.6	R	This study
H	141	Zhangjiakou, Hebei	pyroclastic rock & tuffaceous sandstone	40.7	115.8	104s	80.4	244.1	3.2	35.3	F&R	This study
I	140	Beipiao, Liaoning	sandstone	41.55 ~ 41.75	120.75 ~ 120.83	21S	82.7	208.6	4.3	40.9	F&R	Ren et al. [41]
J	130	Luanping, Hebei	volcanic rocks & sandstone	40.87 ~ 41.61	117.28 ~ 120.83	35S	80.5	197.4	2.3	42.5	F&R	Ren et al. [41]

Abbreviations: *N*, number of specimens (*s*) or sites (*S*) used for calculation; *Slat.*/*Slong.*, latitude/longitude of sampling site; *Plat.*/*Plong.*, latitude/longitude of paleomagnetic pole; A_{95}, 95% confidence limit; *F*, fold test; *R*, reversal test; *B*, backed test. *, poles calculated with directions after inclination shallowing correction. ^, poles were not used to rebuild APWP. Paleolatitude is calculated at 41°N, 121°E as the reference point.

References

1. Zhai, M.-G., Santosh, M. The early Precambrian odyssey of the North China Craton: a synoptic overview. *Gondwana Res.* **20**, 6-25 (2011).

2. Zhao, G. Sun, M., Wilde, S. A., Sanzhong, L. Late Archean to Paleoproterozoic evolution of the North China Craton: key issues revisited. *Precambrian Res.* **136**, 177-202 (2005).

3. Huang, B., Yan, Y., Piper, J. D. A., Zhang, D., Yi, Z., Yu, S., Zhou, T. Paleomagnetic constraints on the paleogeography of the East Asian blocks during Late Paleozoic and Early Mesozoic times. *Earth-Sci. Rev.* **186**, 8-36 (2018).

4. Zhao, P., Appel, E., Xu, B., Sukhbaatar, T. First Paleomagnetic Result From the Early Permian Volcanic Rocks in Northeastern Mongolia: Evolutional Implication for the Paleo-Asian Ocean and the Mongol-Okhotsk Ocean. *J. Geophys. Res.: Solid Earth* **125**, e2019JB017338 (2020).

5. Zhao, P., Chen, Y., Xu, B., Faure, M., Shi, G., Choulet, F. Did the Paleo-Asian Ocean between North China Block and Mongolia Block exist during the late Paleozoic? First paleomagnetic evidence from central-eastern Inner Mongolia, China. *J. Geophys. Res.: Solid Earth* **118**, 1873-1894 (2013).

6. Faure, M., Lin, W., Monié, P., Le Breton, N., Poussineau, S., Panis, D., Deloule, E. Exhumation tectonics of the ultrahigh-pressure metamorphic rocks in the Qinling orogen in east China: New petrological-structural-radiometric insights from the Shandong Peninsula. *Tectonics* **22**, 1018 (2003).

7. Yang, Z. & Besse, J. New Mesozoic apparent polar wander path for south China: Tectonic consequences. *J. Geophys. Res.: Solid Earth* **106**, 8493-8520 (2001).

8. Zhu, G., Wang, Y., Wang, W., Zhang, S., Liu, C., Gu, C., Li, Y. An accreted micro-continent in the north of the Dabie Orogen, East China: Evidence from detrital zircon dating.

Tectonophysics **698**, 47-64 (2017).

9. Hao, W., Zhu, G., Zhu, R. Timing of the Yanshan Movement: evidence from the Jingxi Basin in the Yanshan fold-and-thrust belt, eastern China. *Int. J. Earth Sci.* **108**, 1961-1978 (2019).

10. Hao, W., Zhu, R., Zhu, G. Jurassic tectonics of the eastern North China Craton: Response to initial subduction of the Paleo-Pacific Plate. *Geol. Soc. Am. Bull.* **133**, 19-36 (2020).

11. Yu, H., Zhang, Z., Shuai, G., Chen, Y., Tang, W. SHRIMP and LA-ICP-MS U-Pb ages and geological significance of the volcanic rocks in the Tiaojishan Formation in Ming Tombs area—Western Hills, Beijing. *Geological Review* **62**, 807-826 (2016).

12. Zhao, Y., Xu, G., Zhang, S.-H. Yanshanian movement and conversion of tectonic regimes in East Asia. *Front. Earth. Sci.* **11**, 319-328 (2004).

13. Xu, H. & Liu, Y.-Q. Jurassic–Cretaceous Transition Terrestrial Red Beds in Northern North China and Their Regional Paleogeography, Paleoecology, and Tectonic Evolution. *Acta Geosci. Sin.* **38**, 25-28 (2017).

14. Xu, H., Liu, Y.-Q., Kuang, H.-W., Liu, Y.-X., Peng, N. Jurassic–Cretaceous terrestrial transition red beds in northern North China and their implication on regional paleogeography, paleoecology, and tectonic evolution. *Palaeoworld* **26**, 403-422 (2017).

15. Xu, H., Liu, Y.-Q., Kuang, H.-W., Jiang, X.-J., Peng, N. U–Pb SHRIMP age for the Tuchengzi Formation, northern China, and its implications for biotic evolution during the Jurassic–Cretaceous transition. *Palaeoworld* **21**, 222-234 (2012).

16. Cope, T. D. Sedimentary evolution of the Yanshan fold-thrust belt, Northeast China. *Ph.D. Thesis*, Stanford University, Califonia, pp. 1–230 (2003).

17. Zhang, H., Wang, M., Liu, X. Constraints on the upper boundary age of the Tiaojishan Formation volcanic rocks in West Liaoning-North Hebei by LA-ICP-MS dating. *Chin. Sci. Bull.*

53, 3574-3584 (2008).

18. Ma, Q. & Xu, Y.-G. Magmatic perspective on subduction of Paleo-Pacific plate and initiation of big mantle wedge in East Asia. *Earth-Sci. Rev.* **213**, 103473 (2021).

19. Chu, Z., et al. High-precisionU-Pb geochronology of the Jurassic Yanliao Biota from Jianchang (western Liaoning Province, China): Age constraints on the rise of feathered dinosaurs and eutherian mammals, *Geochem. Geophys. Geosyst.* **17**, 3983–3992 (2016).

20. Roberts, A. P., Cui, Y. & Verosub K. L. Wasp-waisted hysteresis loops: Mineral magnetic characteristics and discrimination of components in mixed magnetic systems. *J. Geophys. Res.: Solid Earth* **100**, 17909-17924 (1995).

21. McElhinny, M. W. Statistical Significance of the Fold Test in Palaeomagnetism. *Geophys. J. R. Astron. Soc.* **8**, 338-340 (1964).

22. Watson, G. S. & Enkin, R. J. The Fold Test in Paleomagnetism as a Parameter-Estimation Problem. *Geophys. Res. Lett.* **20**, 2135-2137 (1993).

23. Mcfadden, P. L. & Mcelhinny M. W. Classification of the Reversal Test in Paleomagnetism. *Geophys. J. Int.* **103**, 725-729 (1990).

24. Tauxe, L., Kylstra, N. & Constable, C. Bootstrap statistics for paleomagnetic data. *J. Geophys. Res. Solid Earth* **96**, 11723-11740 (1991).

25. Kent, D. V. & Irving, E. Influence of inclination error in sedimentary rocks on the Triassic and Jurassic apparent pole wander path for North America and implications for Cordilleran tectonics. *J. Geophys. Res. Solid Earth* **115**, 103 (2010).

26. Iglesia Llanos, M. P., lanza, R., Riccardi, A. C., Geuna, S., Laurenzi, M. A., Ruffini, R. Palaeomagnetic study of the El Quemado complex and Marifil formation, Patagonian Jurassic igneous province, Argentina. *Geophys. J. Int.* **154**, 599-617 (2003).

27. Hargraves, R., Rehacek, J. & Hooper, P. R. Palaeomagnetism of the Karoo igneous rocks in southern Africa. *S. Afr. J. Geol.* **100**, 195-212 (1997).

28. Van Fossen, M. C., & Kent, D. V. A palaeomagnetic study of 143 Ma kimberlite dikes in central New York State. *Geophys. J. Int.* **113**, 175-185 (1993).

29. Halvorsen, E. A paleomagnetic pole position of Late Jurassic/Early Cretaceous dolerites from Hinlopenstretet, Svalbard, and its tectonic implications. *Earth Planet. Sci. Lett.* **94**, 398-408 (1989).

30. Kulakov, E. V., Torsvik, T. H., Doubrovine, P. V., Slagstad, T., Ganerød, M., Silkoset, P., Werner, S. C. Jurassic fast polar shift rejected by a new high-quality paleomagnetic pole from southwest Greenland. *Gondwana Res.* **97**, 240-262 (2021).

31. Kent, D. V., Kjarsgaard, B. A., Gee, J. S., Muttoni, G. & Heaman L. M. Tracking the Late Jurassic apparent (or true) polar shift in U-Pb-dated kimberlites from cratonic North America (Superior Province of Canada). *Geochem. Geophys. Geosyst.* **16**, 983-994 (2015).

32. Besse, J. & Courtillot, V. Apparent and true polar wander and the geometry of the geomagnetic field over the last 200 Myr. *J. Geophys. Res.* **107**, 2300 (2002).

33. Torsvik, T. H., et al. Phanerozoic polar wander, palaeogeography and dynamics. *Earth-Sci. Rev.* **114**, 325-368 (2012).

34. Muttoni, G., Dallanave, E. & Channell J. E. T. The drift history of Adria and Africa from 280 Ma to Present, Jurassic true polar wander, and zonal climate control on Tethyan sedimentary facies. *Palaeogeogr., Palaeoclimatol., Palaeoecol.* **386**, 415-435 (2013).

35. Fu, R. R. , Kent, D. V., Hemming, S. R., Gutierrez, P. & Creveling, J. R. Testing the occurrence of Late Jurassic true polar wander using the La Negra volcanics of northern Chile. *Earth Planet. Sci. Lett.* **529**, 115835 (2020).

36. Muttoni, G. & Kent D. V. Jurassic Monster Polar Shift Confirmed by Sequential Paleopoles From Adria, Promontory of Africa. *J. Geophys. Res. Solid Earth* **124**, 3288-3306 (2019).
37. Liu, S., Lin, C., Liu, X., Zhuang, Q. Syn-tectonic sedimentation and its linkage to fold-thrusting in the region of Zhangjiakou, North Hebei, China. *Sci. China: Earth Sci.* **61**, 681-710 (2018).
38. Uno K. & Huang B. Constraints on the Jurassic swing of the apparent polar wander path for the North China Block. *Geophys. J. Int.* **154**, 801-810 (2003).
39. Gao, Y., Zhang, S., Zhao, H., Ren, Q., Yang, T., Wu, H., Li, H. North China block underwent simultaneous true polar wander and tectonic convergence in late Jurassic: New paleomagnetic constraints. *Earth Planet. Sci. Lett.* **567**, 117012 (2021).
40. Yi, Z. Y., Liu, Y. Q. & Meert, J. G. A true polar wander trigger for the Great Jurassic East Asian Aridification. *Geology* **47**, 1112-1116 (2019).
41. Ren, Q. et al. New Late Jurassic to Early Cretaceous paleomagnetic results from North China and southern Mongolia and their implications for the evolution of the Mongol-Okhotsk suture. *J. Geophys. Res. Solid Earth* **123**, 10,370-310,398 (2018).
